# Deoxyribonuclease DNASE1L3 promotes innate immunity against DNA virus infection by enhancing the cGAS-STING pathway

**DOI:** 10.3389/fimmu.2026.1806552

**Published:** 2026-10-23

**Authors:** Min Wang, Yilan Wang, Jian Ni, Yujie Zhan, Menglan Bao, Xiyang Wang, Qingsong Shen, Xuechun Zhang, Kailing Pan, Xianguo Chen, Zan Huang, Min Peng

**Affiliations:** 1 College of Life Sciences, Hubei Key Laboratory of Cell Homeostasis, Department of Oncology of Renmin Hospital, Wuhan University, Wuhan, China; 2 Department of Cardiology of Renmin Hospital, Wuhan University, Wuhan, China; 3 Department of Oncology of Renmin Hospital, Wuhan University, Wuhan, China; 4 Department of Cardiothoracic Surgery, Jinhua Municipal Central Hospital, Affiliated Jinhua Hospital of Zhejiang University School of Medicine, Jinhua, China

**Keywords:** cGAS-STING pathway, deoxyribonuclease, DNASE1L3, HSV-1, innate immunity

## Abstract

Deoxyribonucleases are known to scavenge self-DNA to dampen innate immune responses and prevent autoimmune diseases. Whether and how deoxyribonucleases respond to DNA virus infection are largely elusive. In this study, HSV-1 infection significantly upregulated and activated DNASE1L3. *In vitro*, DNASE1L3 overexpression upregulated the production of type I interferons and pro-inflammatory cytokines, enhanced IRF3 phosphorylation, and dampened viral replication whereas DNASE1L3 deficiency caused the opposite phenotypes. *In vivo*, *Dnase1l3* knockout impaired innate immunity against HSV-1 and led to an early onset of death and low survival rate of mice. Mechanistically, DNASE1L3 enhanced the activation of cGAS-STING pathway through novel mechanisms. On one hand, DNASE1L3 bound to STING, enhanced its oligomerization and the recruitment of TBK1 independent of the nuclease activity. On the other hand, DNASE1L3 facilitated the release of HMGB1, and cGAS from chromatin dependent on the nuclease activity, which ultimately augmented cGAS oligomerization and cGAMP production. Our study revealed novel roles of DNASE1L3 and the underlying mechanisms coordinating innate immune responses to self-DNA and viral DNA.

## 1 Introduction

Innate immune responses to DNA are tightly associated with multiple pathophysiological conditions including autoimmune diseases, DNA virus infection, and tumor immunity, and the activation of cGAS-STING pathway plays essential roles in these processes (1, 2). Physiologically, self-DNA has to be cleared or tolerated whereas microbe DNA activates innate immune signaling to fight pathogenic infection. Chromatin-bound cGAS is inactive but reactivated by viral infection (3), which suggests a structural basis for differential innate immune responses to DNA in different biological processes. However, mechanisms coordinating distinct innate immune responses to DNA in different biological processes remain to be further clarified.

DNA degradation by deoxyribonucleases is an effective way for the body to prevent autoimmune diseases (4). Among them, DNASE1, DNASE1L1, and DNASE1L3 are mainly responsible for clearing extracellular DNA, while DNASE1L2, TREX1, and PLD3/PLD4 are mainly responsible for clearing intracytoplasmic DNA (5). Mutations in these genes are closely correlated to systemic lupus erythematosus (SLE), systemic sclerosis, and rheumatoid arthritis (5, 6). Deficiencies of these genes cause defects in clearing autologous DNA that leads to sustained activation of the STING signaling, endolysosome TLR7/9 pathway, or RIG-I-MAVS pathway (5, 6). Of note, DNASE1L3 is structurally characterized by nuclear localization signals and a C-terminal domain, which confer DNASE1L3 of nuclear localization as well as the ability to cleave nucleosomal and liposomal DNA extracellularly and intracellularly (6, 7). DNASE1L3 mutations or decreased activity associated with systemic lupus erythematosus and rheumatoid arthritis (8, 9), and DNASE1L3 knockout mice mimic these phenotypes by inducing B cell differentiation and the expression of type I interferons in plasmacytoid dendritic cells via endosomal TLR7/9 (10–12). However, whether and how DNASE1L3 may regulate innate immunity in DNA virus infection remains unclear.

In this study, we found that DNASE1L3 was upregulated and activated by herpes simplex virus 1 (HSV-1) that in turn dampened HSV-1 infection and promoted mice survival *in vitro* and *in vivo*. DNASE1L3 did so by promoting STING oligomerization in cytoplasm and cGAS reactivation in nucleus. Our study unveils unexpected roles of DNASE1L3 and the underlying mechanisms in innate immune response to DNA virus infection, which are opposite to its function in dampening innate immune response via endosomal TLR7/9 pathway to prevent self-DNA-induced autoimmune diseases.

## 2 Materials and methods

### 2.1 Mice

*Dnase1l3^−/−^* mice (C57BL/6 background) were generated using the CRISPR/Cas9 technique and *Dnase1l3* gene knockout was confirmed as previously reported (13). All animal experiments were approved by the Animal Care and Use Committee of the College of Life Sciences of Wuhan University. Care was provided following the criteria outlined in the Guide for The Care and Use of Laboratory Animals prepared by the National Academy of Sciences and published by the National Institutes of Health.

### 2.2 Cell culture

THP-1 cells (ATCC, Cat#TIB-202) were cultured in complete RPMI 1640 medium (Gibco, Cat#31800022). L929 (ATCC, Cat#CCL-1), 143B (ATCC, Cat#CVCL-2270), and Vero (ATCC, Cat# CCL-81) cells were maintained in complete DMEM medium (Gibco, Cat#12100046). HEK293T cells were kindly provided by Dr. Bo Zhong from Wuhan University, which retain the ability to response to HSV-1 infection and DNA transfection for dual-luciferase assay (14–16). Primary mouse embryo fibroblasts (MEFs) were prepared from *E14.5* embryos and cultured in complete DMEM medium. To culture bone marrow-derived macrophages (BMDMs), bone marrow cells were isolated from femurs of wildtype (*Wt*) and *Dnase1l3^−/−^* (*KO*) mice and cultured in complete DMEM medium supplemented with 20% of L929-conditioned medium. All complete medium contained FBS (10%, vol/vol) (Cegrogen, Cat#A0500-3010) and streptomycin–penicillin (1%, vol/vol) (HyClone, Cat# SV30010). All cells were incubated at 37°C in a 5% CO_2_ and 90% humidity atmosphere.

For transient transfection of HEK293T cells, polyethyleneimine (Polyscience, Cat#24765) was used according to the manufacturer’s protocol. To establish stable overexpression or knockdown cells, THP-1 cells were infected with lentivirus in the presence of polybrene (10 μg/mL) and selected with puromycin (10 μg/mL) for 5 days as previously described (17). Sequences of sgRNAs specific for *DNASE1L3* were listed in **Supplementary Table S2** and the knockdown efficiency was verified by Western blotting.

### 2.3 Cell model and mouse model of herpes simplex virus infection

Cells were infected with HSV-1 (5×10^6^ PFU per 1×10^6^ cells) for one hour. The supernatants were removed and cells were washed twice with pre-warmed PBS followed by culture in full medium for 16 hours. Viral replication was analyzed by quantitative RT-PCR. To infect mouse, age- and sex-matched *Wt* and *Dnase1l3^−/−^* mice were injected with HSV-1 (1×10^7^ PFU per mouse) through tail vein and the survival of animals was monitored every day as previously described (14). Mice were sacrificed and the liver, spleen, lung, brain or serum were collected for quantitative RT-PCR analysis, Western blotting analysis, or ELISA at the indicated time points post infection.

### 2.4 Plaque formation assay

The supernatants of HSV-1-infected BMDMs or THP-1 cells were collected and used to infect monolayers of Vero cells. One hour after infection, the supernatants were removed and the infected Vero cells were washed with pre-warmed PBS twice followed by incubation with complete DMEM medium supplemented with 1% methylcellulose for 36–48 hours. Cells were fixed with 4% paraformaldehyde for 15 minutes and stained with 1% crystal violet for 15 minutes before counting the plaques.

### 2.5 Measurement of deoxyribonuclease activity

THP-1 and HEK293T cells were washed and lysed in PBS containing 0.2% NP40. To examine the effect of PV6R on deoxyribonuclease activity, PV6R (100 μM) was added to the culture medium 6 hours prior to sample collection. The deoxyribonuclease activity in the cell lysates or cell culture supernatants was assayed by using genomic DNA or nucleosomal DNA from 143B human osteosarcoma cells, which do not express DNASE1L3 and possess stable chromatin structure, uniform nucleosome assembly, and abundant Alu repetitive sequences. Therefore, genomic DNA from 143B cells have been adopted as a standard substrate for studying DNase1, DNASE1L3, and CAD family nuclease due to such unique characters (8, 18, 19). In brief, 5 μL of the deoxyribonuclease sample was applied to the genomic DNA or nucleus solution (derived from 1×10^5^ 143B cells in a reaction buffer containing 25 mM HEPES, 1 mM MgCl_2_, 1 mM CaCl_2_, 100 μg/ml BSA with total reaction volume of 30 μL). After incubation for 30 minutes at room temperature, EDTA was added to a final concentration of 10 mM to stop the reaction. DNA fragmentation was assessed by agarose gel electrophoresis, and the Alu200/Alu76 ratio was measured by quantitative PCR. A lower ratio indicates greater DNA fragmentation and higher nuclease activity.

### 2.6 Subcellular fractionation of cytoplasmic, nuclear-soluble, and chromatin-bound constituents

HEK293T and THP-1 cells (5×10^6^) were collected. Cytoplasmic fraction, nuclear-soluble fraction, and chromatin-bound fraction were prepared as previously described (14) and used for Western blotting. Detailed protocol was provided in Supplemental information and data.

### 2.7 Dual luciferase reporter assay

HEK293T cells were co-transfected with firefly luciferase-expressing reporter vectors and renilla luciferase-expressing inner control vector in combinations of vectors overexpressing different genes as indicated (14). After culture for 48 hours, cells were harvested and lysed in lysis buffer for 30 minutes at room temperature. A luciferase kit (Promega, Cat#E1980) and Promega Max20/20 was used to measure the firefly and renilla luciferases activities following manufactory manual.

### 2.8 Quantitative RT-PCR

Total RNA was extracted with TRIzol (Vazyme, Cat#R411-01), reverse-transcribed with All-in-One RT SuperMix Perfect (Vazyme, #R333-01), and used for PCR with SYBR qPCR Master Mix (Vazyme, #Q311-02) following manufactory instructions. PCR was performed in the following conditions: 95 °C for 2 min followed by 95 °C for 15 s and 60 °C for 30 s for 40 cycles. The reactions were run in triplicates on the Quantagene q225 Real-Time PCR System (Kubo Tech). The relative quantitation of real-time PCR product was measured using the comparative ΔΔCT method and presented in bar graph format. The sequences of primer sets were listed in **Supplementary Table S1** in Supplemental information and data.

### 2.9 Co-immunoprecipitation and Western blotting

Co-immunoprecipitation and Western blotting were performed as previously described (17, 20). Antibodies used in this study were listed in the Supplemental information and data.

### 2.10 Semi-denaturing detergent agarose gel electrophoresis

SDD-AGE was performed as previously described (14, 21). In brief, cells were collected and lysed for 30 minutes with lysis buffer (20 mM Tris–HCl, pH 7.4-7.5, 150 mM NaCl, 1 mM EDTA, 1% NP-40) containing inhibitors for proteases and phosphatases. After centrifugation for 10 minutes at 12,000×g, supernatants were collected and added with 1×Sample buffer (0.5×TBE, 10% glycerol, 2% SDS, and 0.0025% bromophenol blue) and loaded onto a vertical 1.5% agarose gel. After electrophoresis in the running buffer (1×TBE and 0.1% SDS) for 60 minutes with a constant voltage of 100 V at 4°C, the proteins were subjected to Western blotting analysis.

### 2.11 ELISA

The IFN-β and IL-6 in serum were determined by ELISA kits (JONLBIO, Cat#JL20219, Cat#JL20268) following manufactory manual.

### 2.12 Immunofluorescence assay

Cells were fixed with 4% paraformaldehyde (PFA) in PBS for 10 min at room temperature. Cells then were blocked and permeabilized for 30 minutes in blocking buffer (PBS containing 1% bovine serum albumin (BSA) and 0.1% Triton X-100). Cells were then incubated with primary antibody at 4 °C overnight and secondary antibody for 1 hour at room temperature. Nuclei were labeled by staining with DAPI. Images were collected using a Leica SP8 confocal laser microscopy system (Leica Microsystems) and processed with LAS X (V3.7.4.23463). All images are representative of at least three independent experiments.

### 2.13 Detection of cGAMP by mass spectrometry

Standard cGAMP was purchased from Topscience (Cat#T10065L). The cGAMP levels in samples were measured by liquid-mass spectrometry (Vanquish Core HPLC and Q Exactive HF-X MS Orbitrap from Thermo Fisher). Detailed protocol was provided in Supplemental information and data.

### 2.14 Statistical analysis

All data were presented as the mean ± SEMs and analyzed by GraphPad software. Data are representative of ≥3 independent biological replicates. Differences between experimental and control groups were determined by Student’s *t*-test (where two groups of data were compared) or by one-way or two-way ANOVA analysis (where more than two groups of data were compared). The *P* value <0.05 was defined as statistical significance. For animal survival analysis, the Kaplan–Meier method was adopted to generate the survival curves and analyzed with log-rank. The statistical analysis method used in each experiment was specified in the figure legends.

## 3 Results

### 3.1 DNASE1L3 upregulation by HSV-1 infection enhances innate immunity *in vitro*

Nucleases and nucleic acid editing enzymes play key roles in DNA sensing and autoimmune diseases (4). However, whether and how these enzymes may regulate DNA virus infection is largely unknown. In this study, cell lysates from HSV-1-infected THP-1 cells exhibited stronger nuclease activities to degrade naked genomic DNA isolated from 143B cells than that from mock-infected THP-1 cells evidenced by the reduction of Alu200/Alu76 ratio (the ratio of long 200-bp Alu sequence to short 76-bp Alu sequence amplified by quantitative PCR) (**Figure 1A**; the corresponding agarose gel electrophoresis is shown in **Supplementary Figure S1A**). This result suggests that HSV-1 infection increases the total intracellular nuclease activity. Consistently, HSV-1 infection upregulated the expression of multiple nuclease genes in human cell lines and primary mouse cells, of which DNASE1L3 (D1L3) was consistently upregulated in all cells (**Supplementary Figures S1B–E**). To test how these genes might contribute to HSV-1-induced innate immune responses, HEK293T cells were employed to perform dual luciferase reporter assay, which retained modest response to HSV-1 infection and DNA transfection for dual-luciferase assay described in previous reports (14–16). TREX1 was shown to reduce the HSV-1-induced activity of *IFNB* gene promoter as previously reported (22, 23). Interestingly, DNASE1L1 (D1L1), DNASE1L2 (D1L2), and DNASE1L3 (D1L3) promoted the activation of *IFNB* gene promoter, of which DNASE1L3 displayed the most significant effect and exerted function dose-dependently (**Figures 1B, C**). Indeed, cell lysates from HSV-1-infected THP-1 cells showed increased ability to digest the nucleosomal DNA isolated from 143B cells (**Figure 1D**; the corresponding agarose gel electrophoresis is shown in **Supplementary Figure S1F**), which is an optimal substrate for DNASE1L3 but not for other nucleases as a unique character of DNASE1L3. Furthermore, DNASE1L3 enhanced the activity of *ISRE* and *NF-κB* reporters (**Figure 1E**). These observations strongly suggest that DNASE1L3 upregulation is tightly associated with HSV-1 infection and enhances innate immune response. The endogenous protein expression of DNASE1L3 in THP-1 and HEK293T cells was detected and multiple bands were observed as previously reported, which was further verified by CRISPR/Cas9-mediated *DNASE1L3* knockout (**Supplementary Figure S1G, H**). DNASE1L3 overexpression showed lower molecular weight than the endogenous DNASE1L3 in HEK293T cells as expected (13). It has been speculated that the multiple bands of DNASE1L3 originate from cell-type-specific differences in post-translational modifications such as ADP-ribosylation, proteolytic cleavage, or secretory processing in these cell lines (13, 24–28). Moreover, only low-molecular-weight DNASE1L3 was secreted outside the THP-1 cells and measurable low-molecular-weight DNASE1L3 was observed in cytoplasm and nucleus in DNASE1L3-overexpressing THP-1 cells upon HSV-1 infection (**Supplementary Figure S1I**). DNASE1L3 overexpression promoted HSV-1-induced IRF3 phosphorylation, enhanced the expression of *IFNB*, *TNFA*, and *IL6*, and inhibited HSV-1 replication in THP-1 cells (**Figures 1F–I**). These results demonstrate that upregulation and activation of DNASE1L3 by HSV-1 infection in turn enhances innate immune response.

**Figure 1:**
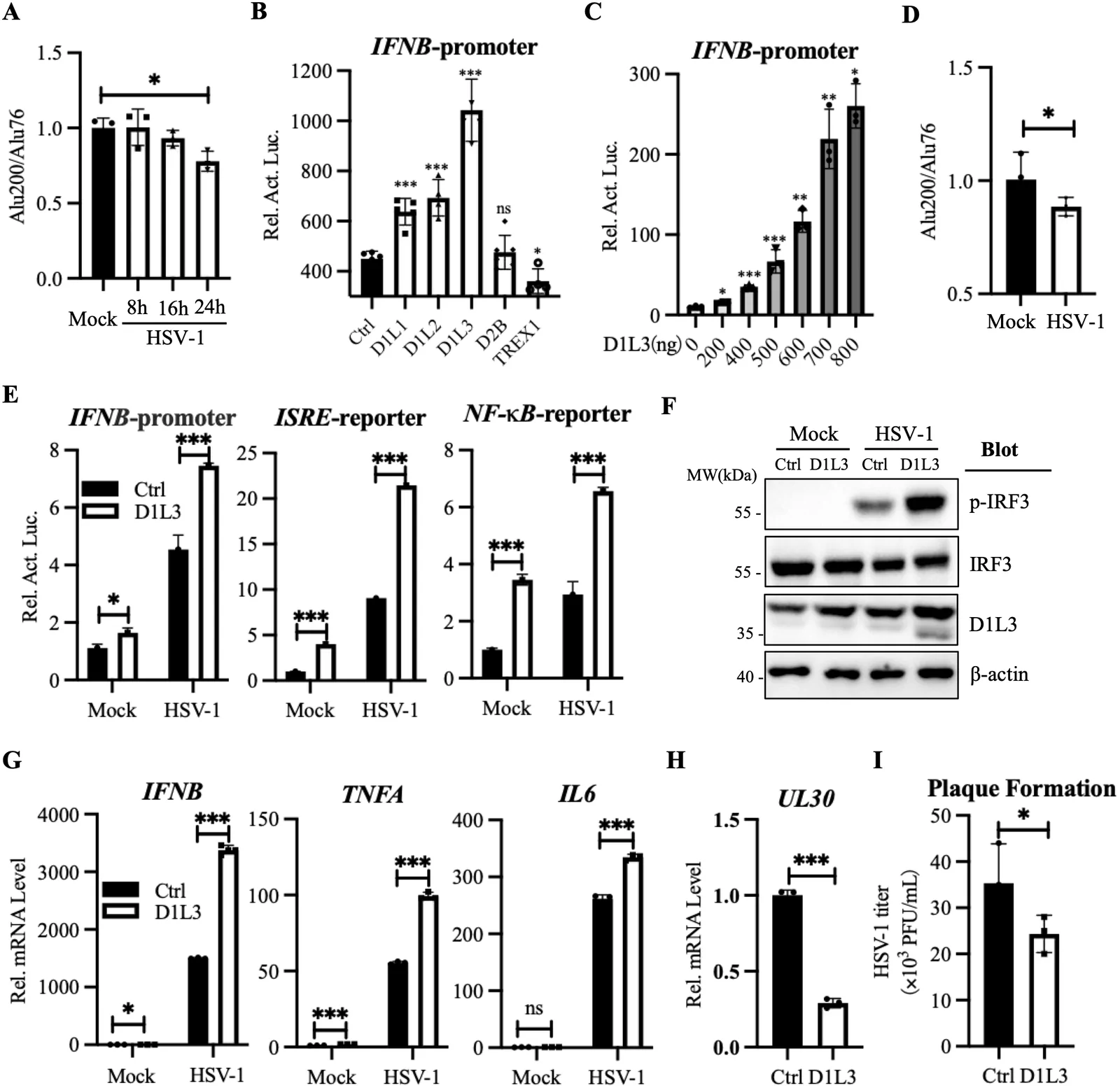
DNASE1L3 upregulation by HSV-1 infection enhances innate immunity *in vitro*. **(A)** Genomic DNA isolated from 143B cells was treated with cell lysates from Mock or HSV-1-infected THP-1 cells for times as indicated. Quantitative PCR was performed to measure the repetitive sequence Alu and the ratio of the long fragment amplification product Alu200 to the short fragment amplification product Alu76 was calculated. **(B)** Analysis on the activity of *IFNB* gene promoter by dual luciferase reporter assay in HSV-1-infected HEK293T cells co-transfected with Ctrl, DNASE1L1- (D1L1), DNASE1L2- (D1L2), DNASE1L3- (D1L3), DNASE2B- (D2B), or TREX1-overexpressing plasmids. **(C)** Analysis on the activity of *IFNB* gene promoter by dual luciferase reporter assay in HEK293T cells transfected with different doses of DNASE1L3-expressing plasmids as indicated. **(D)** The nucleus isolated from 143B cells was treated with cell lysates from Mock or HSV-1-infected THP-1 cells. Quantitative PCR was performed and the Alu200/Alu76 was calculated. **(E)** Analysis on the activity of *IFNB* gene promoter, ISRE reporter, or NF-κB reporter in Mock or HSV-1-infected HEK293T cells transfected with vector (Ctrl) or DNASE1L3-expressing (D1L3) plasmids as indicated. **(F)** Cell lysates from Mock or HSV-1-infected control (Ctrl) or DNASE1L3-overexpressing (D1L3) THP-1 cells were used for Western blotting analysis as indicated. **(G)** Quantitative RT-PCR analysis on the expression of *IFNB*, *TNFA*, and *IL6* in Mock- or HSV-1-infected Ctrl or D1L3 THP-1 cells. **(H)** Quantitative RT-PCR analysis on the expression of *UL30* mRNA to measure HSV-1 replication in HSV-1-infected Ctrl and D1L3 THP-1 cells. **(I)** Plaque formation assay to measure HSV-1 replication using supernatants from HSV-1-infected Ctrl and D1L3 THP-1 cells. *P < 0.05; **P < 0.01; ***P < 0.001; ns, non-significance. Statistical significance was determined using two-tailed Student’s t tests in **(D, H, I)** or one-way ANOVA in **(A–C)**, two-way ANOVA in **(E, G)**.

### 3.2 DNASE1L3 deficiency inhibits innate immunity against HSV-1 *in vitro*

We further tested the effect of DNASE1L3 deficiency on HSV-1-induced innate immune response. Upon HSV-1 infection, DNASE1L3 knockdown (sgD1L3) decreased the phosphorylation of TBK1 and IRF3, inhibited the expression of *IFNB*, *TNFA*, and *IL6*, and promoted the replication of HSV-1 virus in THP-1 cells (**Figures 2A–D**). Furthermore, primary mouse embryo fibroblasts (MEFs) isolated from *Dnase1l3^-/-^* mice (*KO*) exhibited decreases of the expression of *Ifnb*, *Ifna4*, and *Il6* induced by HSV-1 or by DNA90 (cGAS agonist) transfection compared to *Wt* cells (**Figure 2E**; **Supplementary Figure S1J**). Moreover, reintroduction of DNASE1L3 into *KO* MEFs rescued the expression of *Ifnb* (**Figure 2F**). In consistent, primary *KO* bone marrow-derived macrophages (BMDMs) showed similar phenotypes (**Figures 2G–I**). Collectively, these data suggest that DNASE1L3 deficiency dampens innate immunity against HSV-1 *in vitro*.

**Figure 2:**
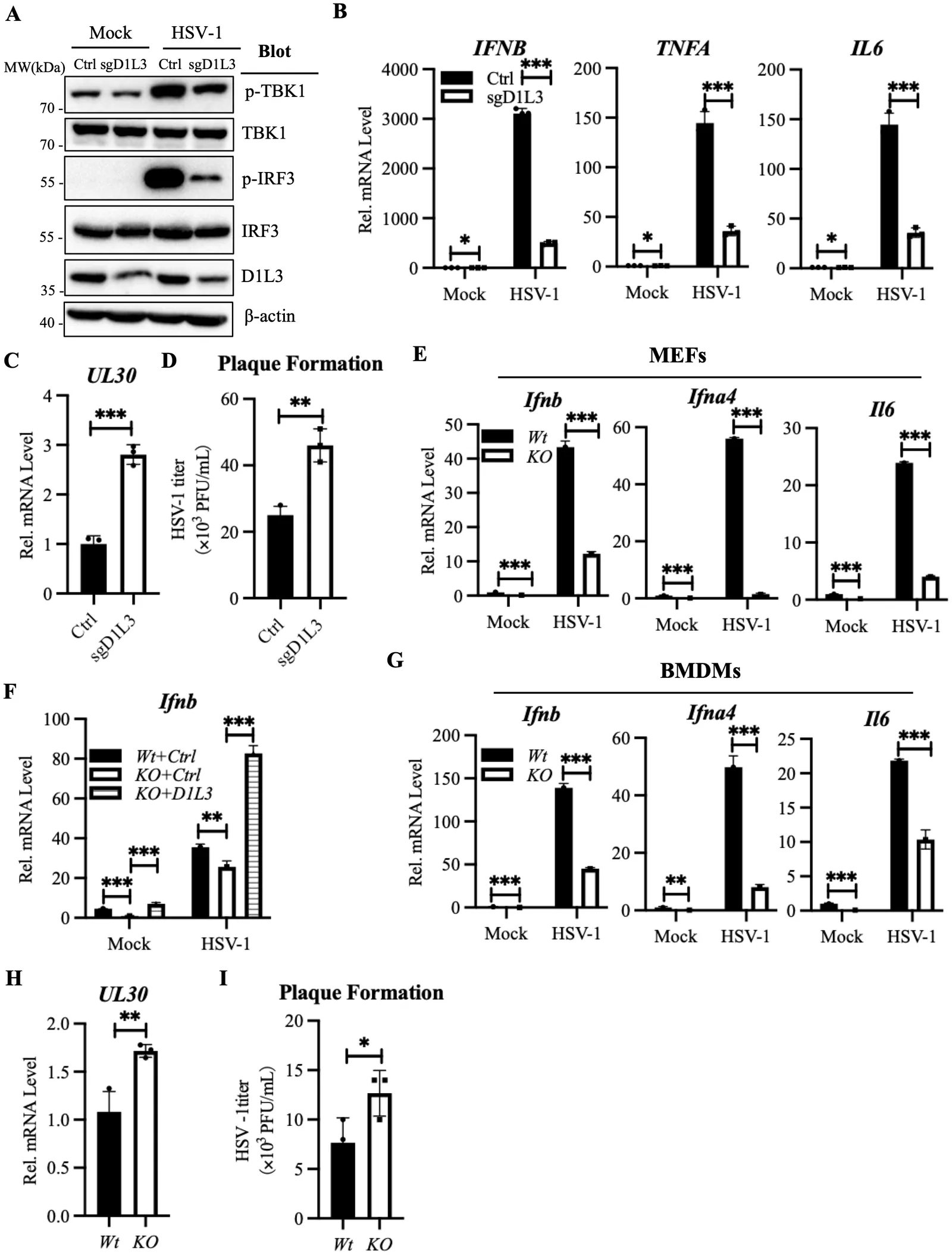
DNASE1L3 deficiency impairs innate immunity against HSV-1 infection *in vitro*. **(A)** Cell lysates from Mock or HSV-1-infected control (Ctrl) or DNASE1L3-knockdown (sgD1L3) THP-1 cells were used for Western blotting analysis as indicated. **(B)** Quantitative RT-PCR analysis on the expression of *IFNB*, *TNFA*, and *IL6* in Mock or HSV-1-infected Ctrl or sgD1L3 THP-1 cells. **(C)** Quantitative RT-PCR analysis on the expression of *UL30* to measure HSV-1 replication in HSV-1-infected Ctrl and sgD1L3 THP-1 cells. **(D)** Plaque assay to measure HSV-1 replication using supernatants from HSV-1-infected Ctrl and sgD1L3 THP-1 cells. **(E)** Quantitative RT-PCR analysis on the expression of *Ifnb*, *Ifna4*, *Il6* in Mock or HSV-1-infected wildtype (*Wt*) and *Dnase1l3^-/-^* (*KO*) MEFs. **(F)** Quantitative RT-PCR analysis on the expression of *Ifnb* in control (*Wt*+Ctrl), *KO* (*KO*+Ctrl), and DNASE1L3-reconstituted KO (KO+D1L3) MEFs. **(G)** Quantitative RT-PCR analysis on the expression of *Ifnb*, *Ifna4*, *Il6* in Mock or HSV-1-infected *Wt* and *KO* BMDMs. **(H)** Quantitative RT-PCR analysis on the expression of *UL30* mRNA to measure HSV-1 replication in HSV-1-infected *Wt* and *KO* BMDMs. **(I)** Plaque formation assay to measure HSV-1 replication using supernatants from HSV-1-infected *Wt* and *KO* BMDMs. *P < 0.05; **P < 0.01; ***P < 0.001; ns, non-significance. Statistical significance was determined using two-tailed Student’s t tests in **(C–D, H–I)** or two-way ANOVA in **(B, E–G)**.

### 3.3 DNASE1L3 enhances antiviral innate immune responses *in vivo*

To examine the role of DNASE1L3 in host defense against HSV-1 infection *in vivo*, *Wt* and *KO* mice were infected by intravenous injection of HSV-1 as previously described (14). The spleen weight/body weight ratio of *KO* mice was greater than that of *Wt* mice whereas comparable values were observed in Mock-infected *Wt* and *KO* mice (**Figure 3A**; **Supplementary Figure S2A**). In consistent, the mRNA levels of *Ifnb*, *Ifna4*, and *Il6* were significantly decreased in lung and liver, and the mRNA levels of viral *ICP0* were significantly increased in the lung and brain from HSV-1-infected KO mice compared to that from HSV-1-infected *Wt* mice (**Figure 3B–D**; **Supplementary Figures S2B, C**). Moreover, levels of IFN-β and IL-6 proteins in the serum from *KO* mice were lower than that from *Wt* mice (**Figure 3E**). Most importantly, the *KO* mice showed an earlier onset of death (day 5 vs. day 9 after HSV-1 infection) and a lower survival rate than *Wt* mice (**Figure 3F**). These data suggest that DNASE1L3 enhances the innate immunity against HSV-1 *in vivo*.

**Figure 3:**
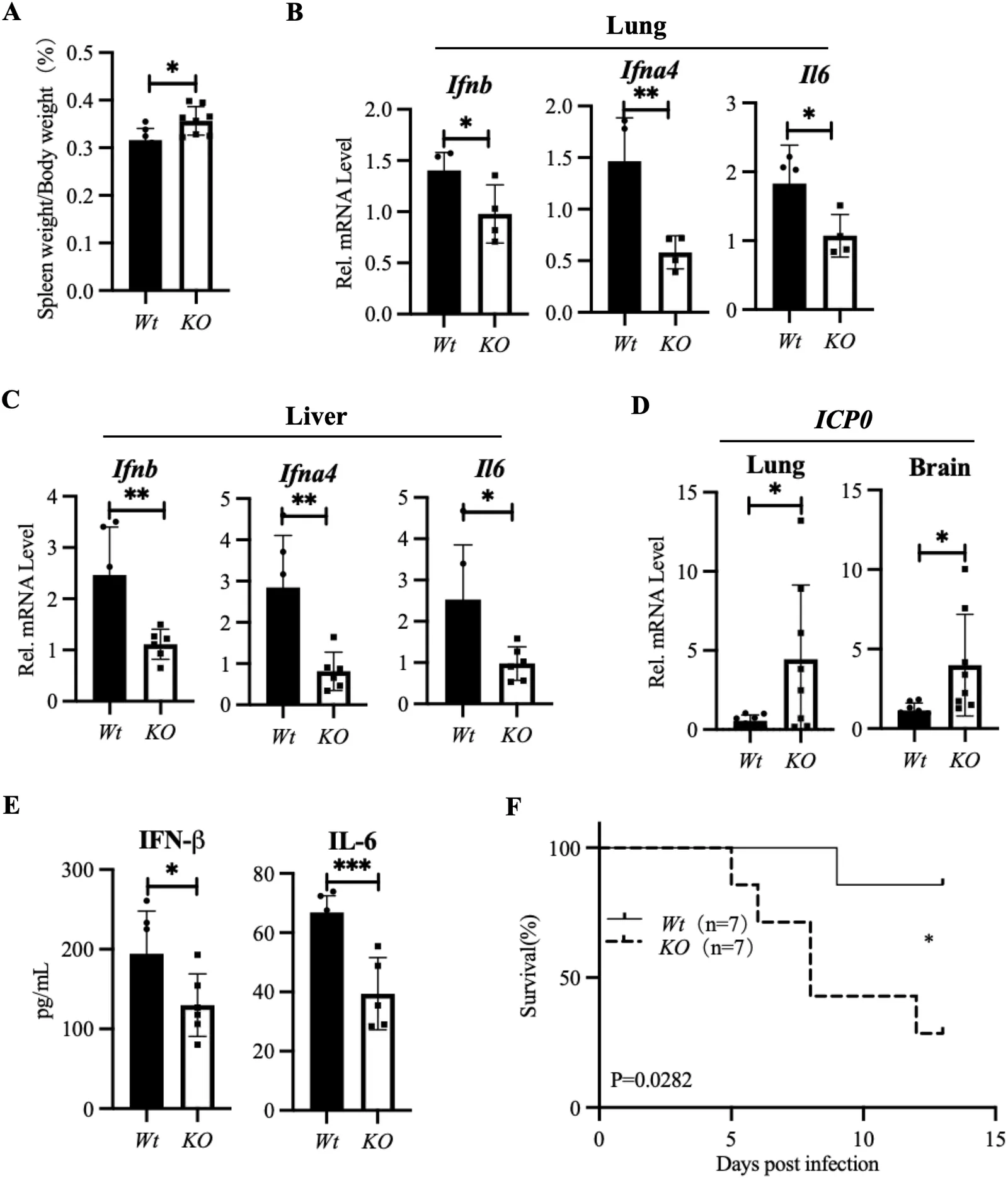
DNASE1L3 enhances antiviral innate immune responses *in vivo*. **(A)** The ratio of spleen weight to body weight of *Wt* (n = 7) and *KO* mice (n = 8) infected with HSV-1 (1×10^7^ PFU per mouse) for 48h. **(B)** Quantitative RT-PCR analysis on the expression of *Ifnb*, *Ifna4*, *Il6* mRNA in the lungs from Wt (n = 4) and KO mice (n = 4) infected with HSV-1 (1×10^7^ PFU per mouse)for 24h. **(C)** Quantitative RT-PCR analysis on the expression of *Ifnb*, *Ifna4*, *Il6* mRNA in the livers from Wt (n = 6) and KO mice (n = 6) infected with HSV-1 (1×10^7^ PFU per mouse) for 48h. **(D)** Quantitative RT-PCR analysis on the expression of *ICP0* to measure the replication of HSV-1 in the lungs and brains from Wt (n = 8) and KO (n = 8) mice infected with HSV-1 (1×10^7^ PFU per mouse) for 3 days. **(E)** ELISA analysis on IFN-β and IL-6 protein levels in the sera from HSV-1-infected *Wt* (n = 5) and *KO* mice (n = 5)) infected with HSV-1 (1×10^7^ PFU per mouse) for 24h. **(F)** Survival curve of HSV-1-infected *Wt* and *KO* mice (n = 7 for each group). *P < 0.05; **P < 0.01; ***P < 0.001; ns, non-significance. Statistical significance was determined using two-tailed Student’s t tests in **(A–E)** and log-rank analysis in **(F)**.

### 3.4 The nuclease activity is required for DNASE1L3 to enhance HSV-1-induced innate immune response

The ability of DNASE1L3 to digest HSV-1 viral particles was tested by using cell lysates or cell culture supernatants isolated from control and DNASE1L3-overexpressing THP-1 cells. Either cell lysates or culture supernatants from DNASE1L3-overexpressing cells did not impair the ability of HSV-1 to form plaques or digest viral genome, both of which showed potent nuclease activities *in vitro* (**Supplementary Figures S3A–F**). These observations suggest that DNASE1L3 did not exert function extracellularly. To test whether the nuclease activity was necessary for its function, mutant DNASE1L3 (H155Q and R206C) with impaired nuclease activity were used (29, 30) (**Supplementary Figure S3G**). These mutants exhibited reduced ability to enhance HSV-1-induced activity of *IFNB* gene promoter compared to wildtype DNASE1L3 (**Figure 4A**). In consistent, wildtype DNASE1L3 largely rescued the impairment of HSV-1 infection-induced *IFNB* expression caused by DNASE1L3 deficiency, while the ability of mutants to rescue the phenotypes was reduced (**Figure 4B**). Furthermore, the treatment of DNASE1L3 inhibitor PV6R suppressed the nuclease activity and impaired the ability of DNASE1L3 to promote HSV-1-induced activity of *IFNB* gene promoter, *IFNB* gene expression, and IRF3 phosphorylation (**Figures 4C–E**; **Supplementary Figure S3H**). In contrast, PV6R treatment showed marginally inhibitory effect in DNASE1L3-deficient cells (**Figures 4F, G**). These data suggest that the nuclease activity is indispensable for DNASE1L3 to enhance HSV-1-induced innate immune response.

**Figure 4:**
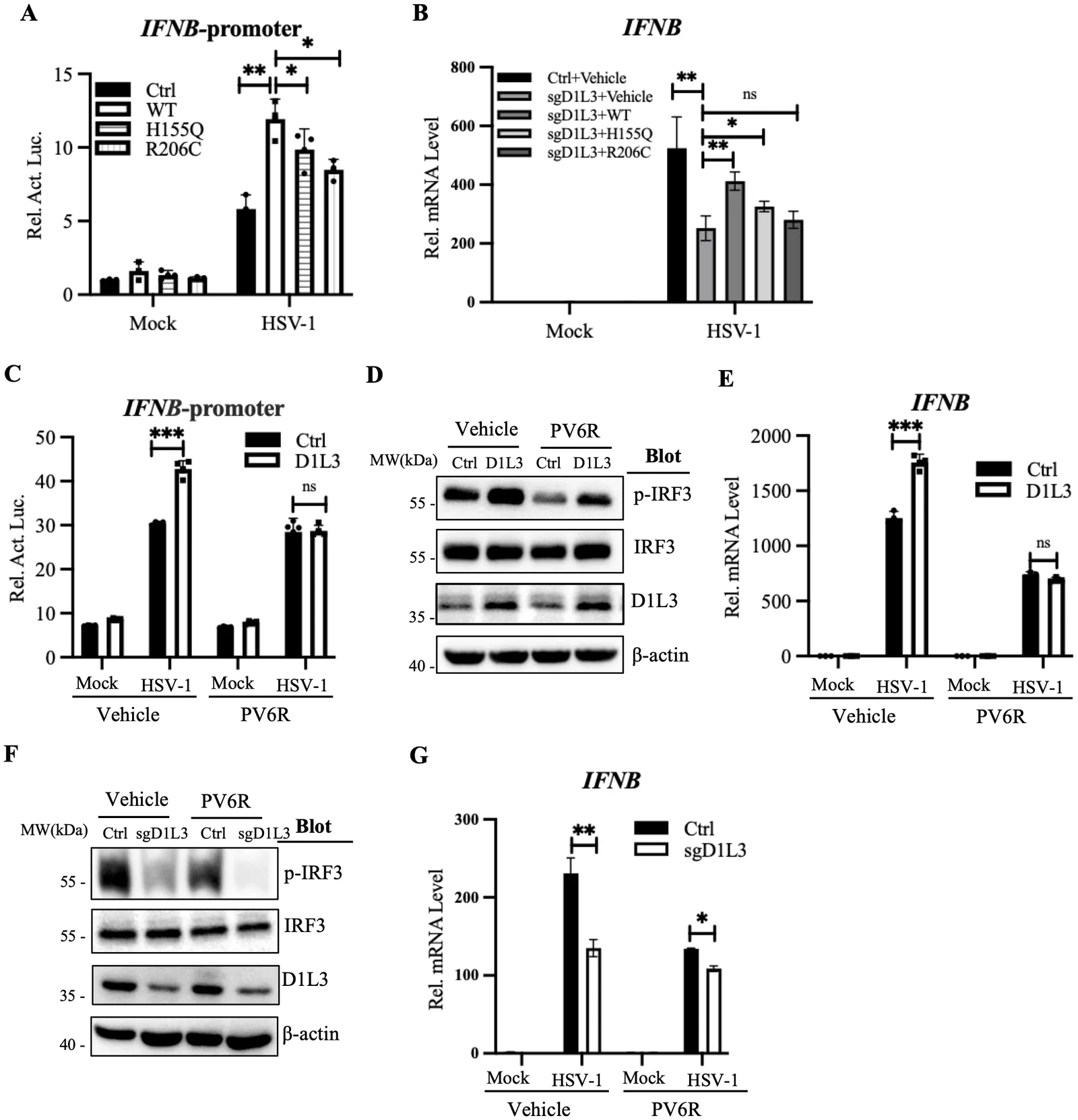
The nuclease activity is required for DNASE1L3 to enhance HSV-1-induced innate immune response. **(A)** The activity of *IFNB* gene promoter was analyzed by dual luciferase reporter assay in Mock and HSV-1-infected HEK293T cells transfected with Ctrl, WT, H155Q, or R206C as indicated. **(B)** Quantitative RT-PCR analysis on the expression of IFNB in control (Ctrl+Vehicle), sgD1L3 (sgD1L3+Vehicle), and DNASE1L3-reconstituted sgD1L3 (sgD1L3+WT, sgD1L3+H155Q, sgD1L3+R206C) THP-1 cells. **(C)** The activity of *IFNB* gene promoter was analyzed by dual luciferase reporter assay in Mock or HSV-1--infected HEK293T cells transfected with Ctrl or D1L3 followed by treatment with Vehicle or PV6R as indicated. **(D)** Cell lysates from HSV-1-infected Ctrl or D1L3 THP-1 cells treated with Vehicle or PV6R were used for Western blotting analysis as indicated. **(E)** Quantitative RT-PCR analysis on the expression of *IFNB* mRNA in Mock and HSV-1-infected Ctrl or D1L3 THP-1 cells treated with Vehicle or PV6R as indicated. **(F)** Cell lysates from HSV-1-infected Ctrl or sgD1L3 THP-1 cells treated with Vehicle or PV6R were used for Western blotting analysis as indicated. **(G)** Quantitative RT-PCR analysis on the expression of IFNB mRNA in Mock and HSV-1-infected Ctrl or sgD1L3 THP-1 cells treated with Vehicle or PV6R as indicated. *P < 0.05; **P < 0.01; ***P<0.001; ns, non-significance. Statistical significance was determined using two-way ANOVA in **(A–C, E, G)**.

### 3.5 DNASE1L3 interacts with STING and enhances its activation

Cell senses viral DNA through cGAS and signaling to STING to induce innate immune response. DNASE1L3 showed the strongest interaction with STING among all signaling molecules and the endogenous DNASE1L3 interaction with STING was verified (**Figure 5A**; **Supplementary Figure S4A**). Immunofluorescence experiment also confirmed the colocalization of DNASE1L3 and STING (**Supplementary Figure S4B**). Domain mapping demonstrated that C-terminal domain deletion (ΔCT) impaired the DNASE1L3 interaction with STING and C-terminal domain of STING (CTD) but not the transmembrane domain of STING (TM) mediated the interaction with DNASE1L3 (**Supplementary Figures S4C, D**). Furthermore, DNASE1L3 significantly enhanced STING-induced *IFNB* promoter activity in the absence and presence of TBK1 (**Supplementary Figure S4E**). TBK1 has been shown to phosphorylate STING and plays a critical role in its activation and oligomerization (31). Indeed, TBK1 increased the amount of DNASE1L3 in the HA-STING immunoprecipitation complex (**Figure 5B**). In the presence of TBK1, DNASE1L3 markedly increased the amount of FLAG-STING and FLAG-TBK1 (solid triangles) in the HA-STING immunoprecipitation complex, and the enhanced STING self-association was observed by co-immunoprecipitation (**Figure 5B**), and increased STING oligomerization was further confirmed by SDD-AGE (**Figure 5C**). More importantly, DNASE1L3 overexpression enhanced HSV-1-induced STING oligomerization (square bracket) whereas DNASE1L3-deficiency showed opposite results in THP-1 cells (**Figures 5D, E**). Noticeably, slow-migrating bands of STING (hollow triangles) were observed and DNASE1L3 increased the intensity of the slow-migrating bands (**Figure 5B**), which might represent the phosphorylated STING. In fact, DNASE1L3 overexpression augmented STING phosphorylation at serine 366 (p-STING) whereas DNASE1L3 knockdown ameliorated STING phosphorylation upon HSV-1 infection in THP-1 cells (**Figures 5F, G**). In support, S366A mutant STING showed weaker interaction with DNASE1L3 than WT STING (**Supplementary Figure S4F**). These observations suggest that DNASE1L3 enhances STING activation and oligomerization by interacting with STING and its phosphorylation appears to be required for the interaction. To further support that DNASE1L3 facilitates STING to recruit TBK1, reciprocal co-immunoprecipitation experiments for STING and TBK1 were performed in Control and DNASE1L3-deficient cells. In either setting, more STING or TBK1 were co-immunoprecipitated in Control cells than that in DNASE1L3-deficient cells (**Supplementary Figures S5A, B**). Immunofluorescence assays revealed stronger colocalization of endogenous TBK1 and STING in control cells than in DNASE1L3-deficient cells (**Supplementary Figure S5C**). These observations strongly demonstrate that DNASE1L3 promotes STING oligomerization and facilitates the recruitment of TBK1 to STING. DNASE1L3 forms a feedforward loop with STING and TBK1 to facilitate phosphorylation and oligomerization of STING, and ultimately enhance innate immune response.

**Figure 5:**
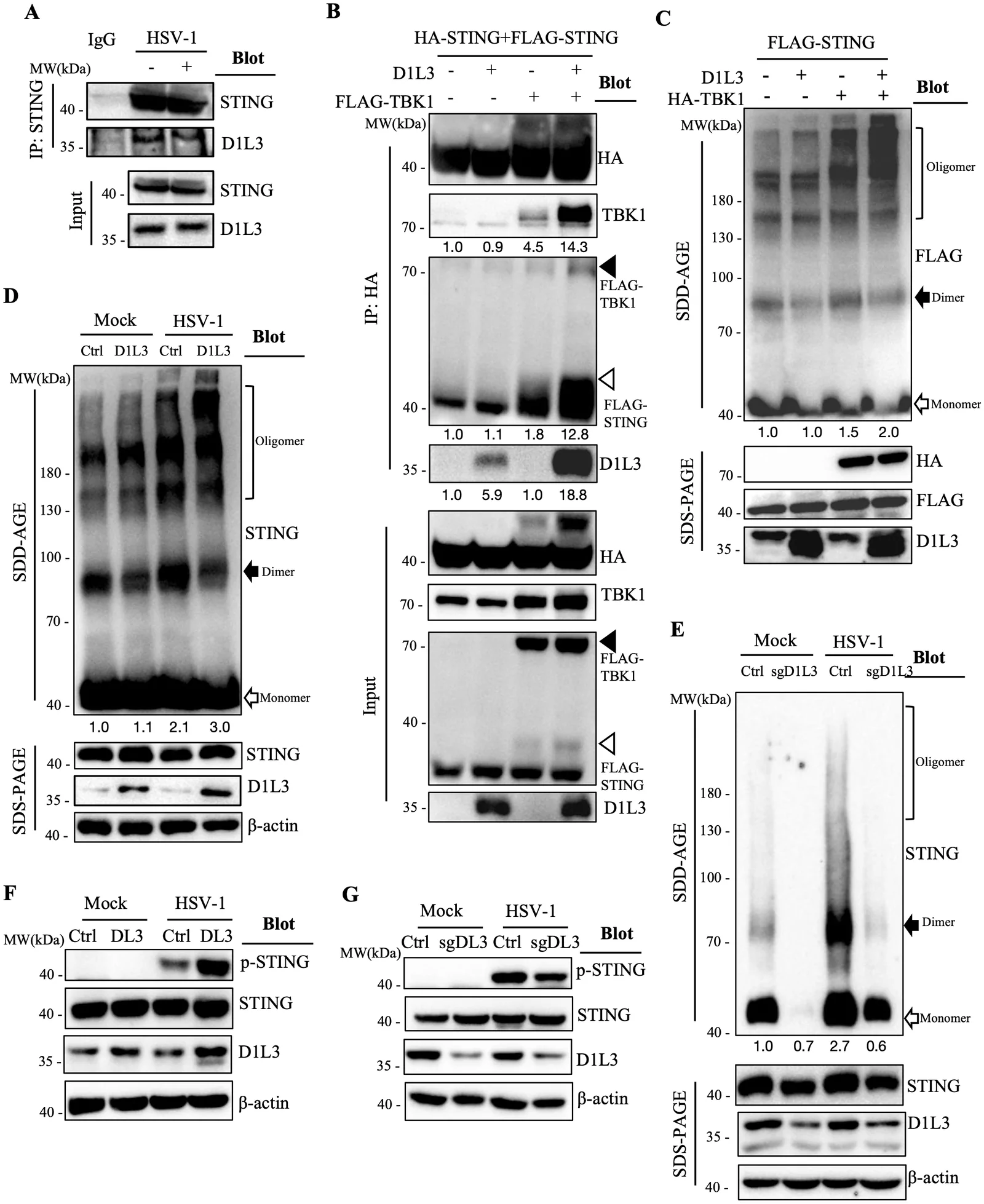
DNASE1L3 interacts with STING and enhances its oligomerization. **(A)** Immunoprecipitation (IgG or anti-STING antibody) followed by Western blotting analysis (with anti-STING or anti-DNASE1L3 antibodies) was performed using Mock- **(-)** and HSV-1-infected (+) THP-1 cells. **(B)** HEK293T cells were transfected with the HA-STING- and FLAG-STING-expressing plasmids in combination with (+) or without **(-)** D1L3- and TBK1-expressing plasmids as indicated. Cell lysates were used for immunoprecipitation with anti-HA antibody and Western blotting analysis was performed. Hollow triangle: shift bands of STING; solid triangle: FLAG-TBK1. **(C)** HEK293T cells were transfected with the FLAG-STING-expressing plasmids in combination with (+) or without **(-)** D1L3- and HA-TBK1-expressing plasmids as indicated. Cell lysates were used for SDD-AGE and SDS-PAGE followed by Western blotting analysis. Square bracket: oligomer; hollow arrow: monomer; solid arrow: dimer. **(D)** Mock or HSV-1-infected Ctrl and D1L3 THP-1 cells were used for SDD-AGE and SDS–PAGE followed by Western blotting analysis. Square bracket: oligomer; hollow arrow: monomer; solid arrow: dimer. **(E)** Mock or HSV-1-infected Ctrl and sgD1L3 THP-1 cells were used for SDD-AGE and SDS-PAGE followed by Western blotting analysis. Square bracket: oligomer; hollow arrow: monomer; solid arrow: dimer. **(F)** Mock or HSV-1-infected Ctrl and D1L3 THP-1 cells were used for Western blotting analysis. **(G)** Mock or HSV-1-infected Ctrl and sgD1L3 THP-1 cells were used for Western blotting analysis. Values beneath the bands indicate relative gray-scale ratios quantified using ImageJ software.

### 3.6 DNASE1L3 enhances TBK1-induced STING oligomerization independent of the nuclease activity

To explore how the nuclease activity might affect DNASE1L3 function, the interaction of H155Q and R206C mutants with STING was tested. Unexpectedly, both H155Q and R206C mutants retained the ability to interact with STING, which was comparable to WT DNASE1L3, and PV6R treatment did not disrupt the interaction (**Figures 6A, B**). In the presence of TBK1, H155Q and R206C mutants also promoted the interaction of HA-STING and FLAG-STING, increased the recruitment of TBK1 (solid triangle), and augmented the intensity of slow-migrating bands (hollow triangle) comparable to WT DNASE1L3 (**Figure 6C**). Alternatively, PV6R treatment did not disrupt the interaction of DNASE1L3 and STING, marginally affected STING oligomerization and the ability to recruit TBK1 (solid triangle), and barely altered the intensity of slow-migrating bands of STING (hollow triangle) (**Figure 6D**). Consistently, H155Q and R206C mutants equally enhanced TBK1-induced activation of *IFNB* gene promoter (**Figure 6E**). PV6R treatment also failed to normalize the enhancement of TBK1-induced activity of *IFNB* gene promoter by DNASE1L3 (**Figure 6F**). To further investigate whether the enhanced TBK1-induced activation of STING by DNASE1L3 relies on its nuclease activity, cGAMP as a potent agonist of STING was used. Under cGAMP stimulation, overexpression of DNASE1L3 enhanced cGAMP-induced *IFNB* expression, whereas knockdown of DNASE1L3 attenuated this response (**Figures 6G, H**). In the presence of cGAMP, treatment with PV6R mildly suppressed *IFNB* expression but did not abolish the promoting effect of DNASE1L3 (**Figure 6I**). These results were in sharp contrast to the effect of PV6R treatment in HSV-1 infection-induced innate immune response (**Figure 4E**). These observations demonstrate that DNASE1L3 nuclease activity appears to be dispensable for its interaction with STING and its ability to enhance TBK1-induced STING aggregation and *IFNB* expression. Considering that nuclease activity is required for DNASE1L3 to enhance HSV-1-induced innate immune response, DNASE1L3 likely also act on the upstream of TBK1 besides promoting STING oligomerization. The overexpression of TBK1 may overwhelm such effect from the upstream of signaling pathway.

**Figure 6:**
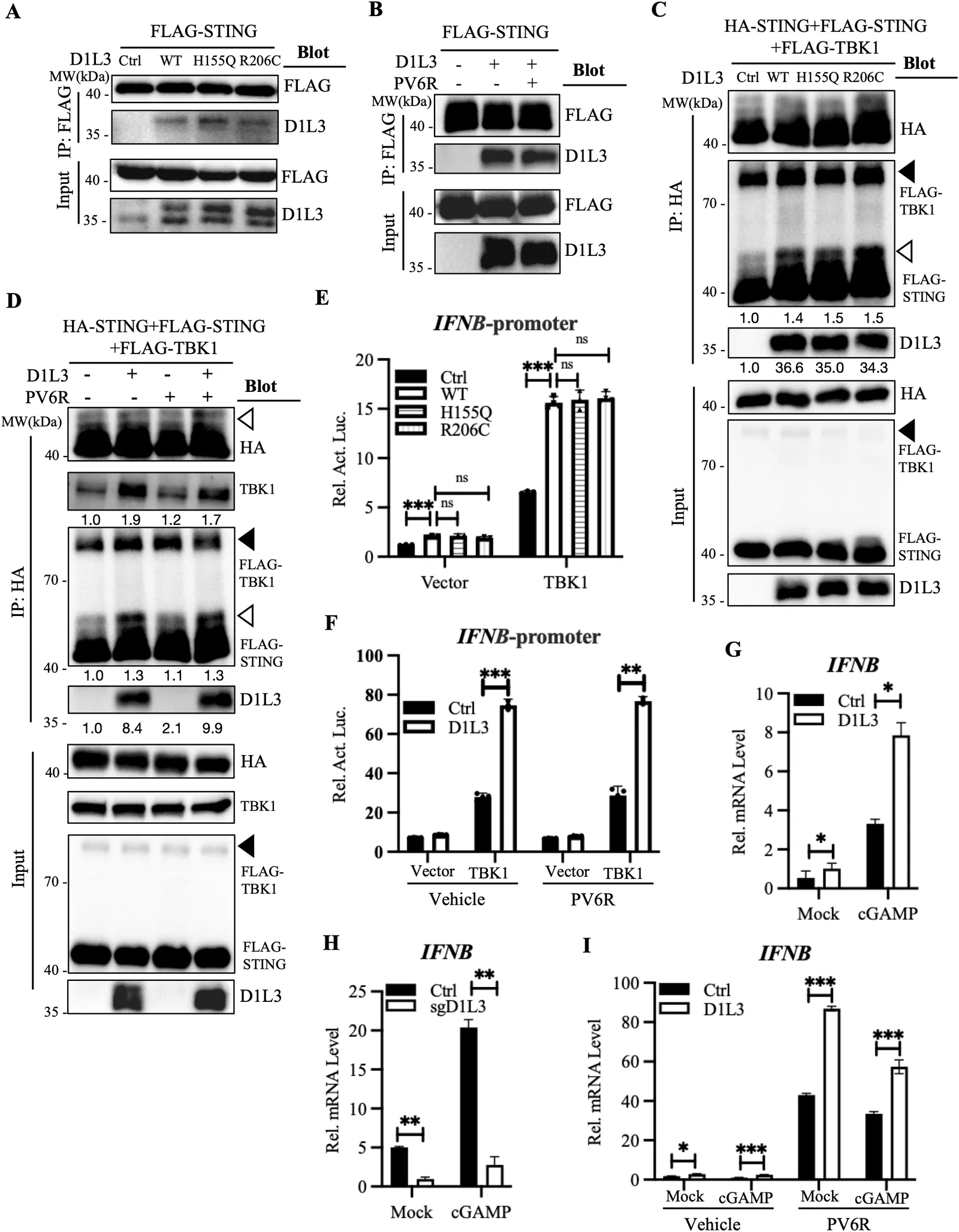
DNASE1L3 enhances TBK1-induced STING oligomerization independent of the nuclease activity. **(A)** HEK293T cells transfected with FLAG-STING in combination with different plasmids as indicated were used for immunoprecipitation with anti-FLAG antibody followed by Western blotting analysis. **(B)** HEK293T cells transfected with FLAG-STING in combination with Vector (-) or D1L3 were treated with Vehicle **(-)** or PV6R (+). Cells were used for immunoprecipitation with anti-FLAG antibody followed by Western blotting analysis. **(C)** HEK293T cells transfected with HA-STING, FLAG-STING, and FLAG-TBK1 in combination with different plasmid as indicated were used for immunoprecipitation with anti-HA antibody followed by Western blotting analysis. Hollow triangle: shift bands of STING; solid triangle: FLAG-TBK1. **(D)** HEK293T cells were transfected with HA-STING, FLAG-STING, and FLAG-TBK1 in combination with Vector **(-)** or D1L3 and treated with Vehicle (-) or PV6R (+). Immunoprecipitation with anti-HA antibody followed by Western blotting analysis was performed. Hollow triangle: shift bands of STING; solid triangle: FLAG-TBK1. **(E)** The activity of *IFNB* gene promoter was analyzed by dual luciferase reporter assay in HEK293T cells transfected with Vector or TBK1 in combination with different plasmids as indicated. **(F)** The activity of *IFNB* gene promoter was analyzed by dual luciferase reporter assay in HEK293T cells transfected with different combinations of Vector, TBK1, Ctrl, and D1L3, and treated with Vehicle or PV6R as indicated. **(G)** Quantitative RT-PCR analysis on the expression of IFNB mRNA in Ctrl or D1L3 THP-1 cells stimulated with mock or cGAMP (STING agonist). **(H)** Quantitative RT-PCR analysis on the expression of IFNB mRNA in Ctrl or sgD1L3 THP-1 cells stimulated with mock or cGAMP (STING agonist). **(I)** Quantitative RT-PCR analysis on the expression of IFNB mRNA in Ctrl or D1L3 THP-1 cells stimulated with mock or cGAMP (STING agonist), and treated with vehicle or PV6R as indicated. Values beneath the bands indicate relative gray-scale ratios quantified using ImageJ software. *P < 0.05; **P < 0.01; ***P < 0.001; ns, non-significance. Statistical significance was determined using two-way ANOVA in **(E–I)**.

### 3.7 DNASE1L3 enhances HSV-1-induced cGAS oligomerization dependent on the nuclease activity

The activation of cGAS and the generation of cGAMP constitute key upstream events of innate immune signaling in response to viral DNA. As expected, DNASE1L3 overexpression augmented whereas DNASE1L3 knockdown decreased HSV-1-induced cGAMP production (**Figures 7A, B**). PV6R treatment normalized the augment of cGAMP production by DNASE1L3 (**Figure 7C**), indicating that DNASE1L3-mediated cGAMP production is dependent on its nuclease activity. Moreover, overexpression of WT DNASE1L3 promoted the cGAS aggregation whereas DNASE1L3 mutants failed to do so (**Figures 7D, E**). PV6R treatment ameliorated the aggregation of FLAG-cGAS and HA-cGAS (**Figure 7F**). Most importantly, DNASE1L3 promoted HSV-1-induced cGAS oligomerization (square bracket) whereas DNASE1L3 deficiency caused the opposite phenotype (**Figures 7G, H**). These data suggest that DNASE1L3 enhances HSV-1-induced cGAS activation and cGAMP production via augmenting cGAS oligomerization, which depended on the nuclease activity.

**Figure 7:**
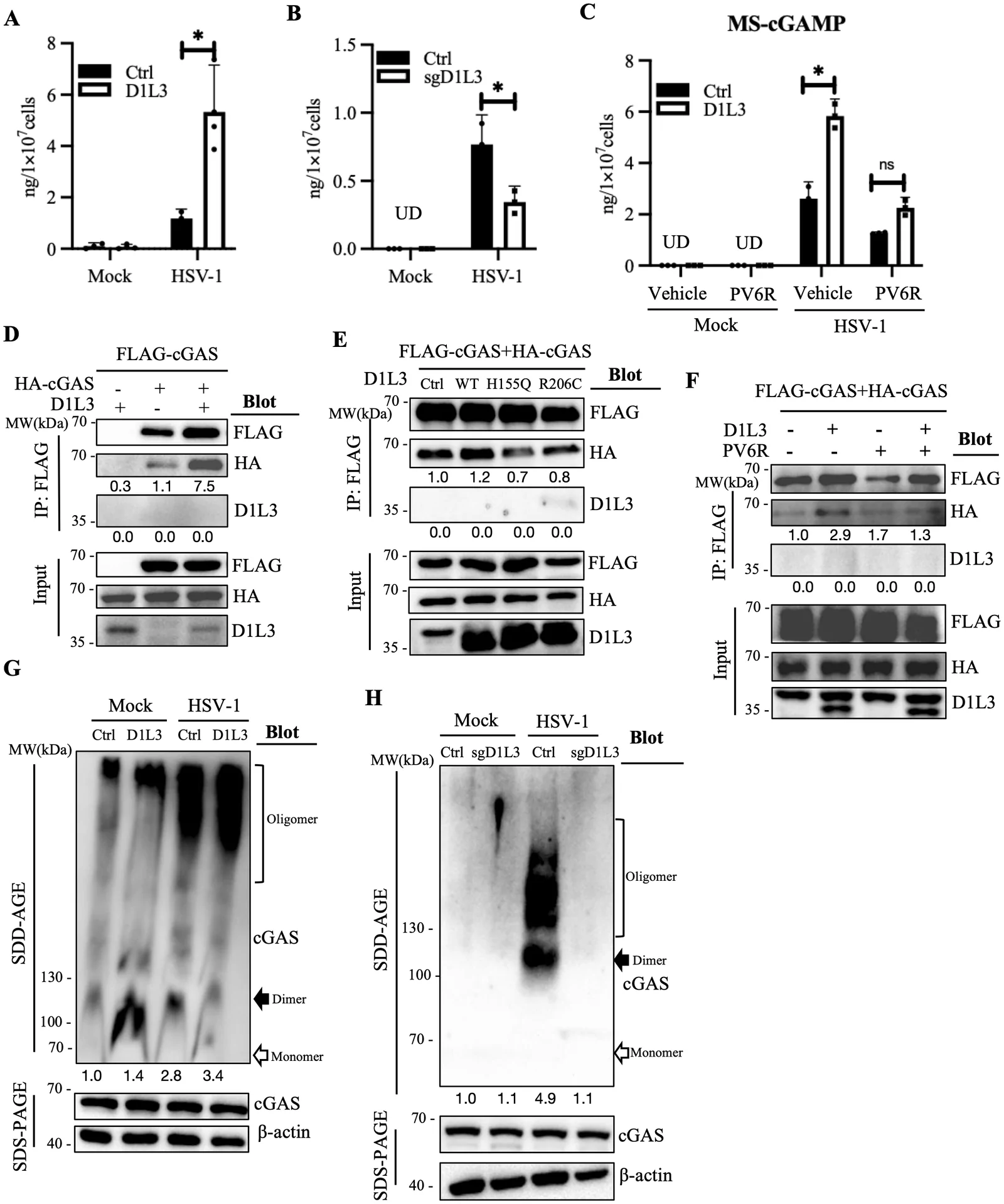
DNASE1L3 enhances HSV-1-induced cGAS oligomerization dependent on the nuclease activity. **(A, B)** Levels of cGAMP were measured by mass spectrometry in Mock or HSV-1-infected Ctrl and D1L3 THP-1 cells **(A)** or Ctrl and sgD1L3 THP-1 cells **(B)**. **(C)** Mock or HSV-1-infected Ctrl and D1L3 THP-1 cells were treated with Vehicle or PV6R as indicated. Levels of cGAMP in these cells were detected by mass spectrometry. **(D)** HEK293T cells transfected with HA-cGAS and FLAG-cGAS in combination with (+) or without (-) D1L3 were used for immunoprecipitation with anti-FLAG antibody followed by Western blotting analysis. **(E)** HEK293T cells transfected with HA-cGAS and FLAG-cGAS in combination with Vector (Ctrl), WT, H155Q, or R206C as indicated were used for immunoprecipitation with anti-FLAG antibody followed by Western blotting. **(F)** HEK293T cells were transfected with HA-cGAS and FLAG-cGAS in combination with Vector (-) or D1L3 and treated with Vehicle (-) or PV6R as indicated. Cells were used for immunoprecipitation with anti-FLAG antibody followed by Western blotting analysis. **(G)** Mock- or HSV-1-infected Ctrl and D1L3 THP-1 cells were used for SDD-AGE and SDS-PAGE followed by Western blotting analysis. Square bracket: oligomer; hollow arrow: monomer; solid arrow: dimer. **(H)** Mock- or HSV-1-infected Ctrl and sgD1L3 THP-1 cells were used for SDD-AGE and SDS-PAGE followed by Western blotting analysis. Square bracket: oligomer; hollow arrow: monomer; solid arrow: dimer. Values beneath the bands indicate relative gray-scale ratios quantified using ImageJ software. *P < 0.05; **P < 0.01; ***P < 0.001; ns, non-significance. Statistical significance was determined using two-way ANOVA in **(A–C)**.

### 3.8 DNASE1L3 facilitates the release of cGAS, HMGB1, and H1.2 from chromatin

Both cGAS and DNASE1L3 have been shown to function in cytoplasm and nucleus, and DNASE1L3 may be activated by histone H1 (32). Moreover, the nuclease activity of DNASE1L3 is closely associated with the release of HMGB1 (33), which is known to activate cGAS (34, 35). Indeed, DNASE1L3 overexpression significantly increased the nuclease activity in nucleus but not in cytoplasm upon HSV-1 infection (**Figure 8A**; the corresponding agarose gel electrophoresis is shown in **Supplementary Figure S6A**), suggesting that DNASE1L3 may enhance cGAS activation in the nucleus. Chromatin-bound cGAS is inactive but functions normally in immune activation upon release from chromatin (3). Therefore, it is likely that DNASE1L3 may promote cGAS activation by facilitating the release of cGAS, HMGB1 and H1. To test this, the cytoplasmic, nuclear-soluble, and chromatin-bound fractions were separated and the protein levels were measured. In HEK293T cells, DNASE1L3 overexpression increased the protein levels of HMGB1 and H1.2 in nuclear-soluble constituents (**Supplementary Figure S6B**). In THP-1 cells without HSV-1 infection, DNASE1L3 overexpression did not significantly alter the protein levels of cGAS, HMGB1, and H1.2 among these fractions. In contrast, HSV-1 infection increased the protein levels of DNASE1L3, cGAS, HMGB1, and H1.2 in nuclear-soluble fractions in both Ctrl and D1L3-overexpressing THP-1 cells while no significant alterations were found in the cytoplasmic fractions (**Figure 8B**). In contrast, DNASE1L3 knockdown attenuated the HSV-1-induced increase of cGAS, HMGB1 and H1.2 in nuclear-soluble fractions of THP-1 cells (**Figure 8C**). Moreover, the elevated protein levels of cGAS, HMGB1, and H1.2 in the nuclear-soluble fractions were compromised in HEK293T cells overexpressing H155Q and R206C mutants (**Figure 8D**). Furthermore, PV6R treatment abrogated the elevation of the protein levels of DNASE1L3, cGAS, HMGB1, and H1.2 in the nuclear-soluble fractions in both THP-1 and HEK293T cells (**Figure 8E**; **Supplementary Figure S6C**). Considering the critical role of HMGB1 in cGAS activation, these findings collectively point to a possibility that HSV-1 infection enhances DNASE1L3 nucleus localization and facilitates cGAS activation in nucleus by promoting the release of cGAS and HMGB1 from the chromatin.

**Figure 8:**
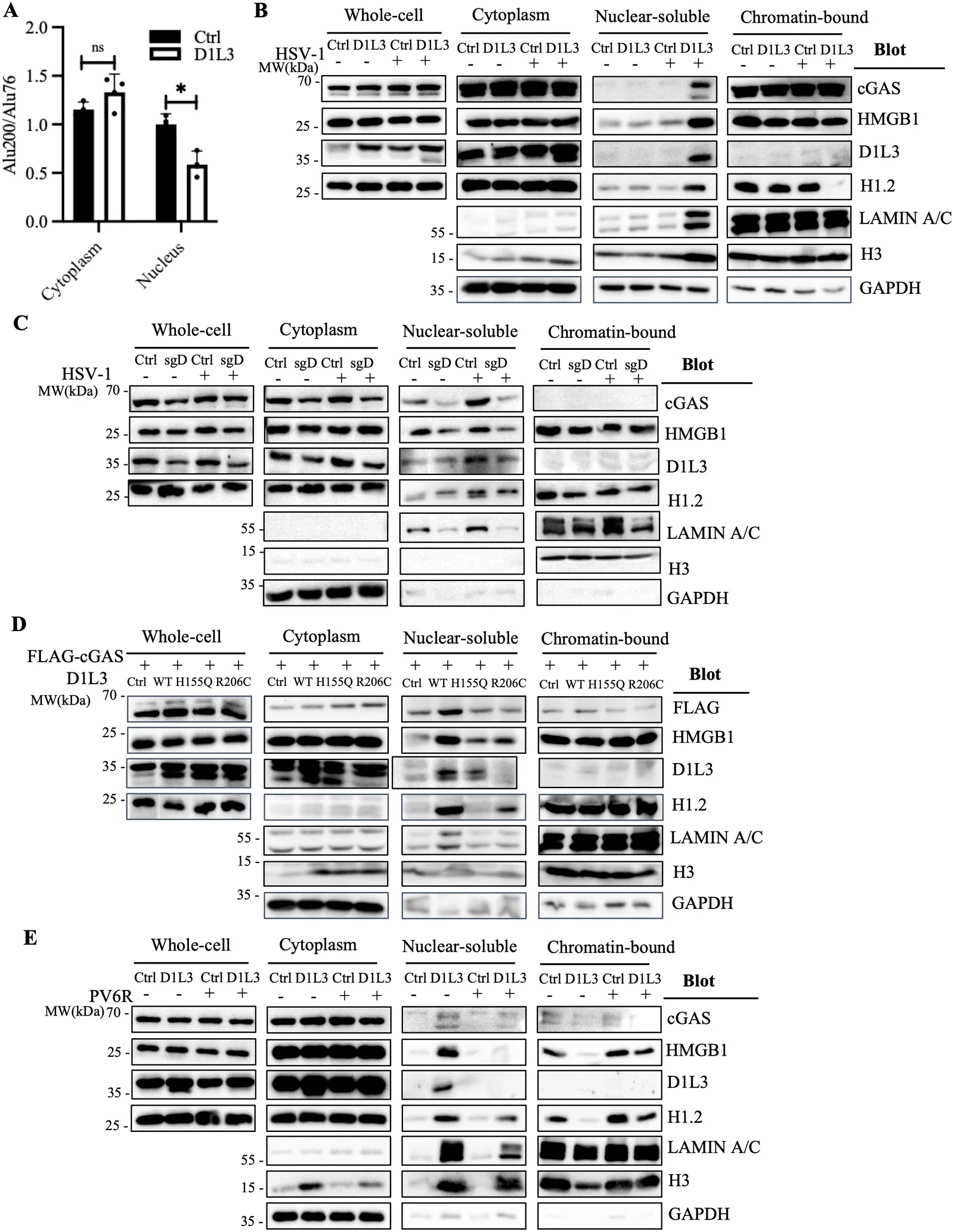
DNASE1L3 facilitates the release of cGAS, HMGB1, and H1.2 from chromatin. **(A)** The cytoplasm and nucleus isolated from HSV-1-infected Ctrl and D1L3 THP-1 cells were used for measurement of nuclease activity as described. *P < 0.05, ns: non-significance. Statistical significance was determined using two-way ANOVA. **(B)** The whole cell lysates as well as cytoplasmic, nuclear-soluble, and chromatin-bound fractions prepared from Mock (-) or HSV-1-infected Ctrl and D1L3 THP-1 cells were used for Western blotting analysis as indicated. **(C)** The whole cell lysates as well as cytoplasmic, nuclear-soluble, and chromatin-bound fractions prepared from Mock (-) or HSV-1-infected Ctrl and sgD1L3 THP-1 cells were used for Western blotting analysis as indicated. **(D)** The whole cell lysates as well as cytoplasmic, nuclear-soluble, and chromatin-bound fractions prepared from HEK293T cells that were transfected with FLAG-cGAS in combination with Vector (Ctrl), WT, H155Q, or R206C were used for Western blotting analysis as indicated. **(E)** HSV-1-infected Ctrl and D1L3 THP-1 cells were treated with Vehicle (-) or PV6R (+) and used to prepare the whole cell lysates as well as cytoplasmic, nuclear-soluble, and chromatin-bound fractions followed by Western blotting analysis.

## 4 Discussion

DNA is one of the important pathogen-associated molecular patterns (PAMPs) that activate innate immune signaling. Extracellular DNASE1L3 has been shown to degrade self-DNA and dampen endosomal TLR7/9 signaling to prevent autoimmune diseases (10, 12). In this study, intracellular DNASE1L3 was shown to promote innate immune signaling and suppress HSV-1 infection, which provided new insights on the different roles of DNASE1L3 in innate immunity.

Nucleases play a crucial role in sensing self-DNA to dampen innate immune responses (5). DNASE1L3 is historically described as a secreted endonuclease that preferentially digests chromatin-packaged DNA rather than naked DNA. Our biochemical assays demonstrate that DNASE1L3 does not directly degrade extracellular HSV-1 viral particles (**Supplementary Figures S3A–F**). Instead, HSV-1 infection induced DNASE1L3 activation in the nucleus (**Figure 8A**). Upon viral entry, HSV-1 DNA enters into the nucleus and assembles into loosely organized viral chromatin. Nuclear-activated DNASE1L3 remodels chromatin to release chromatin-sequestered cGAS, HMGB1 and histone H1.2, potentiating cGAS-dependent sensing. Since most HSV-1 virions deliver genomes into the nucleus, nuclear DNASE1L3 activation may facilitate early host innate immune responses. In addition to its nuclear activity, DNASE1L3 physically interacts with STING in the cytoplasm to promote TBK1 recruitment and STING oligomerization via a nuclease-independent regulatory branch. Thus, DNASE1L3 augments anti-HSV-1 immunity through distinct nuclear and cytoplasmic mechanisms. Notably, our data demonstrate that the nuclease-dependent nuclear function predominates during authentic HSV-1 infection, while nuclease-independent STING potentiation acts downstream of cGAS-STING priming. How DNASE1L3 is upregulated and activated by HSV-1 infection remains to be investigated. The nuclease activity of DNASE1L3 has been shown to be stimulated under apoptotic conditions, resulting in the fragmentation of genomic DNA (25). However, no significant apoptosis phenotypes in early HSV-1-infected cells were observed, and caspase inhibitor Z-VAD-FMK did not eliminate the DNASE1L3-enhanced innate immune response (**Supplementary Figures S7A, B**). Another possibility is that HSV-1 infection alters ADP-ribosylation of DNASE1L3 protein, which has been shown to regulate the nuclease activity of DNASE1L3 (25). Whether DNASE1L3 experiences ADP-ribosylation alterations during HSV-1 infection requires further exploration.

DNASE1L3 appears to enhance the cGAS-STING signaling in HSV-1 infection by multiple ways. Viral DNA can be recognized by cGAS to produce cGAMP, which serves as a ligand for STING oligomerization and activation (36). For effective activation, the cGAS dimer needs to bind to two DNA fragments (>45 bp), or binds to a bent U-shape DNA wrapped with HMGB1 (high-mobility group box 1) and TFAM (transcription factor A mitochondria) that form a trapezoidal structure of cGAS2N-DNA2 (34). Notably, DNASE1L3 differs from other nucleases in its substrate preference. It efficiently digests DNA encapsulated within nucleosomes or liposomes, but only weakly degrades naked DNA, leading to partial degradation only (10, 37). Upon HSV-1 infection, viral DNA is released into the nucleus through nuclear pores and rapidly associates with histones to form unstable, loose nucleosome-like structures (38). Such viral DNA represents a suboptimal substrate for DNASE1L3. It is possible that DNASE1L3 may cleave viral DNA into optimal size to enhance cGAS activation. However, excessive viral DNA degradation by nucleases may also dampen viral infection. Thus, the net effect of nucleases in DNA viral infection is the balance of these possibilities.

Mechanistically, DNASE1L3 appears to enhance the cGAS-STING pathway by two means: nuclease activity-dependent cGAS oligomerization and nuclease activity-independent STING oligomerization. Importantly, efficient enhancement of TBK1-induced STING activation by DNASE1L3 requires partial pre-activation of STING, supported by the greatly reduced interaction between DNASE1L3 and the STING S366A mutant (**Supplementary Figure S4F**). Overexpression of TBK1 bypasses upstream signaling, induces spontaneous TBK1 autophosphorylation and STING phosphorylation (39), and markedly strengthens the binding of DNASE1L3 to STING (**Figure 5B**). Under this condition, wild-type and nuclease-mutant DNASE1L3 comparably enhance TBK1-induced STING oligomerization (**Figure 6C**), and PV6R treatment did not disrupt the interaction of DNASE1L3 and STING (**Figure 6D**). We further activated innate immune responses using cGAMP, a specific agonist of STING, and found that DNASE1L3 markedly augmented cGAMP-induced innate immune activation (**Figure 6G**). Together, these data confirm that DNASE1L3-mediated STING activation strongly relies on prior partial STING activation. This also accounts for why PV6R nearly fully abrogates the antiviral effect of DNASE1L3 during HSV-1 infection (**Figures 4D–G**): inhibition of DNASE1L3 nuclease activity impairs upstream cGAS activation, which fails to initiate STING signaling and thus abolishes the STING-potentiating effect of DNASE1L3.HMGB1 release has been shown to alter the conformation of viral DNA and facilitate cGAS activation (34, 40). However, the underlying mechanisms are largely elusive. The nuclease activity of DNASE1L3 is closely associated with the release of HMGB1 (33). Our study showed that DNASE1L3 promoted cGAS and HMGB1 release into the nucleur-soluble fraction during HSV-1 infection. Moreover, viral infection has been shown to regulate host chromatin accessibility (41). In line with this, DNASE1L3 facilitated H1.2 released from chromatin, which has been shown to regulate host chromatin accessibility (35). DNASE1L3 also plays a role in apoptosis and promotes DNA fragmentation (24). Our preliminary experiment showed that nucleosomal DNA isolated from DNASE1L3-overexpressing cells was more vulnerable to degradation by its own DNASE1L3 or by MNase treatment (**Supplementary Figure S7C**). These data suggest that DNASE1L3 likely alters host chromatin accessibility to facilitate release of cGAS and HMGB1. However, the chromatin-release evidence remains to be correlative at the current stage and additional work is required to directly disentangle nuclease-dependent chromatin remodeling versus indirect secondary effects triggered by innate signaling.

Our study still has some limitations. For instance, multiple migrating DNASE1L3 WB bands are observed across different cell types, likely stemming from cell-type-specific proteolytic cleavage and post-translational modifications, as previously documented in published studies (13, 24, 26–28). Definitive identification of each isoform requires mass-spectrometry-based proteomics and systematic mutagenesis. Moreover, the mechanism underlying HSV-1-driven upregulation of DNASE1L3 remains to be resolved. In addition, SDD-AGE oligomerization assays produce smeared high-molecular-weight signals for cGAS and STING, which is an inherent technical feature of this non-denaturing electrophoresis system (14, 42–44). Although the densitometric quantification may support our conclusions in this study, further quantitative assay remains to be developed.

In summary, the differentially spatial localization of DNASE1L3 appears to determine the distinct functions in regulating innate immune signaling in DNA virus infection and autoimmune diseases. The intracellular DNASE1L3 promotes innate immune responses by enhancing the cGAS-STING pathway through multiple ways. DNASE1L3 facilitates cGAS and HMGB1 release from chromatin in the nucleus upon HSV-1 infection that enhance cGAS activation and cGAMP production. It also promotes STING oligomerization in the cytoplasm (**Supplementary Figure S7D**). In contrast, DNASE1L3 antagonizes autoimmune diseases by degrading the extracellular self-DNA. Our study may help understand mechanisms how human body coordinates innate immune responses to self-DNA and viral DNA.

## Data Availability

The data supporting the conclusions of this article are included in the article and its **Supplementary Material**. Further inquiries can be directed to the corresponding authors.
