# Supplementary material for: Deoxyribonuclease DNASE1L3 promotes innate immunity against DNA virus infection by enhancing the cGAS-STING pathway
Source: Front Immunol. 2026 Oct 23;17:1806552. doi: 10.3389/fimmu.2026.1806552 (PMC13739572; doi:10.3389/fimmu.2026.1806552)
Supplement: Supplementary file 1 [file DataSheet1.pdf]

Supplementary Material

1 Materials and methods

1.1 Subcellular fractionation of cytosolic, nucleus, and chromatin-bound components

Re-suspend 5×10^6 cells in 500 µl Extraction Buffer 1(10 mM HEPES pH 7.9, 10 mM KCl, 1.5 mM $MgCl_2$, 0.34 M sucrose, 10% glycerol, 0.2%NP40), followed by incubation on ice for 10 min. The supernatant obtained by centrifugation was the cytoplasmic fraction (6,500g, 5 min). Wash pellet (the nuclei) in 1 ml Extraction Buffer2 (10 mM HEPES pH 7.9, 10 mM KCl, 1.5 mM $MgCl_2$, 0.34 M sucrose, 10% glycerol) by re-suspending the pellet and incubating on ice for 1 min. Spinning again (6,500g, 5 min) and completely remove the supernatant. Lysing the nuclei by re-suspending the nuclei pellet in 500 µl No-salt Buffer (3 mM EDTA, 0.2 mM EGTA) and incubating on a rotator at 4℃ for 30 min. The next step was centrifugation (6,500g, 5 min). The supernatant obtained by this step was the nuclear soluble fraction, and the pellets were chromatin-bound fraction. Re-suspend the pellet in 200 ul of High-Salt Solubilization Buffer (50 mM Tris–Cl pH 8.0, 2.5 M NaCl, 0.05% NP40) and vortex for 2 min. For good dissociation of chromatin-binding proteins from chromatin, chromatin pellets require ultrasonication (30 cycles, 5s/5s, 4℃). The fractions used for Western blot were immediately mixed with the SDS-PAGE sample buffer and boiled at 95℃ for 15 minutes.

1.2 Concentration of cell culture supernatants

Ctrl and D1L3 THP-1 cells were infected with HSV-1 for 4h, after which the cell culture supernatants were collected. The cell culture supernatants were concentrated by centrifugal filters (Sigma, Cat#UFC801096) following manufactory manual. The cell culture supernatants were concentrated 40-fold, and then subjected to Western blotting analysis.

1.3 Co-immunoprecipitation and Western blot

Briefly, plasmids that express the tagged protein were co-transfected into HEK293T cells. At 24 hrs post-transfection, cells were harvested and lysed with IP Lysis Buffer (20mM Tris (pH 7.4), 150mM NaCl, 1mM EDTA, 1% TritonX-100) supplemented with protease inhibitor cocktail (TargetMol, Cat#C0001), phosphatase inhibitor cocktail (Beyotime, Cat#P1082). One-tenth volume of cell lysates were subject to Western blot analysis to detect the expression of target proteins. The rest of lysates were incubated with primary antibody and protein A+G agarose beads (Beyotime, Cat#P2055) overnight at 4°C with low-speed rocking. The beads were washed with IP lysis buffer for three times. After washing, 60 µl of 2×SDS-PAGE sample buffer was added into the tube containing the beads. The immunoprecipitated proteins were eluted from beads after 20 min of boiling. For Western blot analysis, protein separation on 10% polyacrylamide gels and electronic transfer onto PVDF membranes (Millipore) were performed. After blocking, the membranes were incubated with primary antibodies (1:1000) overnight at 4℃ followed by washing and incubation with secondary antibodies labeled with horseradish peroxidase (HRP) for 1 h at room temperature. After washing, an Enhanced ECL Chemiluminescence Detection Kit (Vazyme, Cat#E412) was used and the signals were detected by a ChemiDoc MP Imaging System (Tanon-5200).

1.4 Detection of cGAMP by mass spectrometry

The samples were ground in 1 mL methanol: water (4/1, v/v) at low temperature for 2 min, centrifuged at 4 ℃ at 12000 r/min for 4 min. 400 μL of the supernatant was collected for rotating drying, and redissolved in 30 μL of 0.1% formic acid aqueous solution for liquid chromatography. The ACQUITY UPLC CSH C18 columns (100 mm×2.1 mm; 1.7 μm, Waters) were used for chromatographic separation of the target compounds. Liquid chromatography was performed with 0.1% formic acid aqueous solution in phase A and 0.1% formic acid acetonitrile in phase B. The temperature of the column incubator was 40 °C. The sample tray was set at 15 °C, and the injection volume was 5 μL. The elution gradient was as follows: 0 min: 2% B; 4 min:20% B; 4.01 min: 2% B; 6.5 min: 2% B. The flow rate was 0.4 mL/min. For the mass spectrometry analyses, Ion source parameters were as follows: spray voltage 3200 V, capillary temperature 320 ℃, sheath gas flow rate 30 arb, aux gas flow rate 10 arb, Aux gas heater temperature 400 ℃. The full scan detection (MS1) analysis used the following acquisition parameters: full scan resolution of 60,000; The AGC target was 1×10^6. The maximum IT was 100 ms. The scanning range was set between 500-800 m/z. Secondary scan (MS/MS) parameters: the resolution was 15,000; the target value of AGC was 1×10^5. The maximum IT was 50 ms. TopN selected the four ions with the highest signal in each cycle. The isolation window was 1.0 m/z. Normalized collision energies (NCE) were 10%, 20%, and 30%; The minimum AGC target value was 8×10^3. The intensity threshold was 1.6×10^5. Finally, Thermo Xcalibur Qual Browser software was used for data acquisition and processing. The Concentration measured by the instrument was calculated as the ratio of the peak area of the target compound to the coefficient of the calibration curve, which was expressed as CC (Calculated Concentration, ng/mL). The correction factor for matrix effect and dilution was 0.4, and the sample dissolution volume was 30 μL. The target metabolite content in the sample is expressed as ng/1×10^7 cells or ng/mg. n. d. indicates that no target metabolite was detected in this sample.

1.5 DNA90 transfection

Prepare double-stranded DNA90 transfection complex following the established protocol for cell transfection. Allow the complex to incubate briefly before adding it to the culture medium. Harvest the cells 12-24 hours post-transfection. The sequence of DNA90 is as follows: 5'-TACAGATCTACTAGTGATCTATGACTGATCTGTACATGATCTACATACAGATCTACTAGT GATCTATGACTGATCTGTACATGATCTACA-3'.

1.6 Reagents, antibodies, and constructs

Antibodies or control IgG were purchased from the indicated manufacturer's, including mouse control IgG (Beyotime, Cat#A7028), rabbit control IgG (Beyotime, Cat#A7016), HRP-conjugated goat anti-mouse or rabbit IgG (ABclonal, Cat#AS003 and AS014); mouse anti-FLAG (ABclonal, Cat#AE005), anti-HA (ABclonal, Cat#AE008), anti-β-Actin (ABclonal, #AC026), anti-GAPDH (ABclonal, Cat#A19056), anti-LAMINA/C (Sigma, Cat#SAB4200236), anti-cGAS (SCBT, Cat#sc-515777); Rabbit anti-FLAG (ABclonal, Cat#AE004), anti-HA (ABclonal, Cat#AE105), anti-H3(HUABIO, Cat#ET1701-64), anti-TBK1 (ABclonal, Cat#A3458), anti-p-TBK1 (ABclonal, Cat#AP1026), anti-IRF3(ABclonal, Cat#A19717), anti-p-IRF3 (ABclonal, Cat#AP1412), anti-p65 (HUABIO, Cat#SZ10-04), anti-p-p65 (HUABIO, Cat#SP07-00), anti-STING (Cell Signaling Technologies, Cat#13647), anti-p-STING-S366 (ABclonal, Cat#AP1369), anti-HMGB1(HUABIO, Cat#SA39-03), and anti-DNASE1L3 (GeneTex, Cat#GTX114363). Pontacyl Violet 6R (Cat#P0593) was purchased from TCI Chemicals. Z-VAD-FMK(Cat#T7020) was purchased from Topscience. MNase (Cat#M0247S) was purchased from New England Biolabs. cGAMP(Cat#T10065L) was

purchased from Topscience. The *IFNB* gene promoter, ISRE reporter, NF-κB luciferase reporter, and mammalian expression plasmids for HA-tagged IRF3, P65, IKKα, and IKKβ were previously described. Mammalian expression plasmid pHAGE-DNASE1L1, DNASE1L2, DNASE2B, TREX1, DNASE1L3, DNASE1L3 mutants, LentiV2-sgRNAs targeting DNASE1L3 were constructed by standard molecular biology techniques. The sequences of primer sets were listed in Table S1 and Table S2 in Supplemental information and data. Mammalian expression plasmids for FLAG-tagged cGAS, STING, TBK1, and HA-tagged cGAS, STING, TBK1 were kindly provided by Dr. Bo Zhong. Mammalian expression plasmids for FLAG-tagged cGAS, STING, TBK1, and HA-tagged STING-TM, HA-tagged STING-CTD were kindly provided by Dr. Yu Liu.

2 Supplementary Figures and Tables

2.1 Supplementary Figures

Figure S1

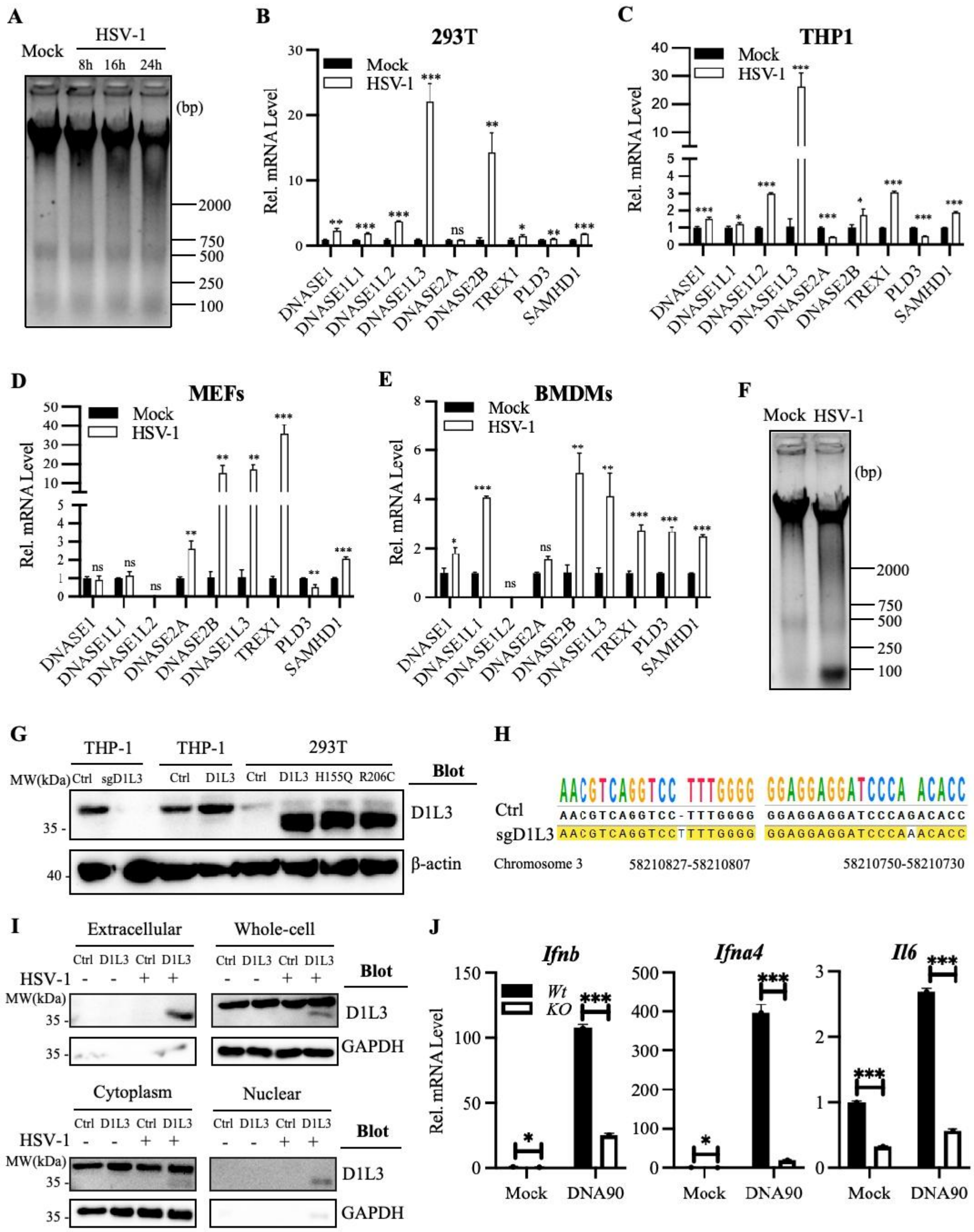

Supplementary Figure 1. DNASE1L3 upregulation by HSV-1 infection enhances innate immunity. (A) This panel shows agarose gel electrophoresis corresponding to the qPCR results in Figure 1A, in which genomic DNA isolated from 143B cells was incubated with cell lysates from Mock- or HSV-1-infected THP-1 cells for the indicated times. **(B-C)** Quantitative RT-PCR analysis on the expression of nuclease genes (*DNASE1*, *DNASE1L1*, *DNASE1L2*, *DNASE1L3*, *DNASE2A*, *DNASE2B*, *TREX1*, *PLD3*, *SAMHD1*) in HEK293T cells (B) and THP-1 cells (C). **(D-E)** Quantitative RT-PCR analysis the expression of nuclease genes (*Dnase1*, *Dnase1l1*, *Dnase1l2*, *Dnase1l3*, *Dnase2a*, *Dnase2b*, *Trex1*, *Pld3*, *Samhd1*) in mouse MEFs (D), mouse BMDMs (E). **(F)**

This panel shows agarose gel electrophoresis corresponding to the qPCR results in Figure 1D, in which the nucleus isolated from 143B cells was treated with cell lysates from Mock or HSV-1-infected THP-1 cells. **(G)** Cell lysates from control (Ctrl) or DNASE1L3-knockdown (sgD1L3) THP-1 cells, control (Ctrl) or DNASE1L3-overexpressing (D1L3) THP-1 cells, HEK293T cells transfected with empty vector (Ctrl), wildtype DNASE1L3 (WT), and mutant DNASE1L3 (H155Q and R206C) were used for Western blotting analysis as indicated. **(H)** Genomic sequencing confirmed the mutations of base pairs in *DNASE1L3* gene in sgD1L3 THP-1 cells. **(I)** Ctrl and D1L3 THP-1 cells were infected with HSV-1 (+) for 4h, after which the cell culture supernatants (Extracellular), total protein (Whole-cell), cytosolic (Cytoplasm), and nuclear (nuclear) fractions were prepared. Proteins from these fractions were then subjected to Western blotting analysis with antibodies to DNASE1L3 or GAPDH. **(J)** Quantitative RT-PCR analysis on the expression of *Ifnb*, *Ifna4*, and *Il6* in wildtype (*Wt*) and *Dnase1l3*$^{-/-}$ (*KO*) MEFs transfected with DNA90(cGAS agonist) for 24h. *: P<0.05; **: P<0.01; ***: P<0.001; ns: non-significance; Statistical significance was determined using two-way ANOVA in (B-E, J).

Figure S2

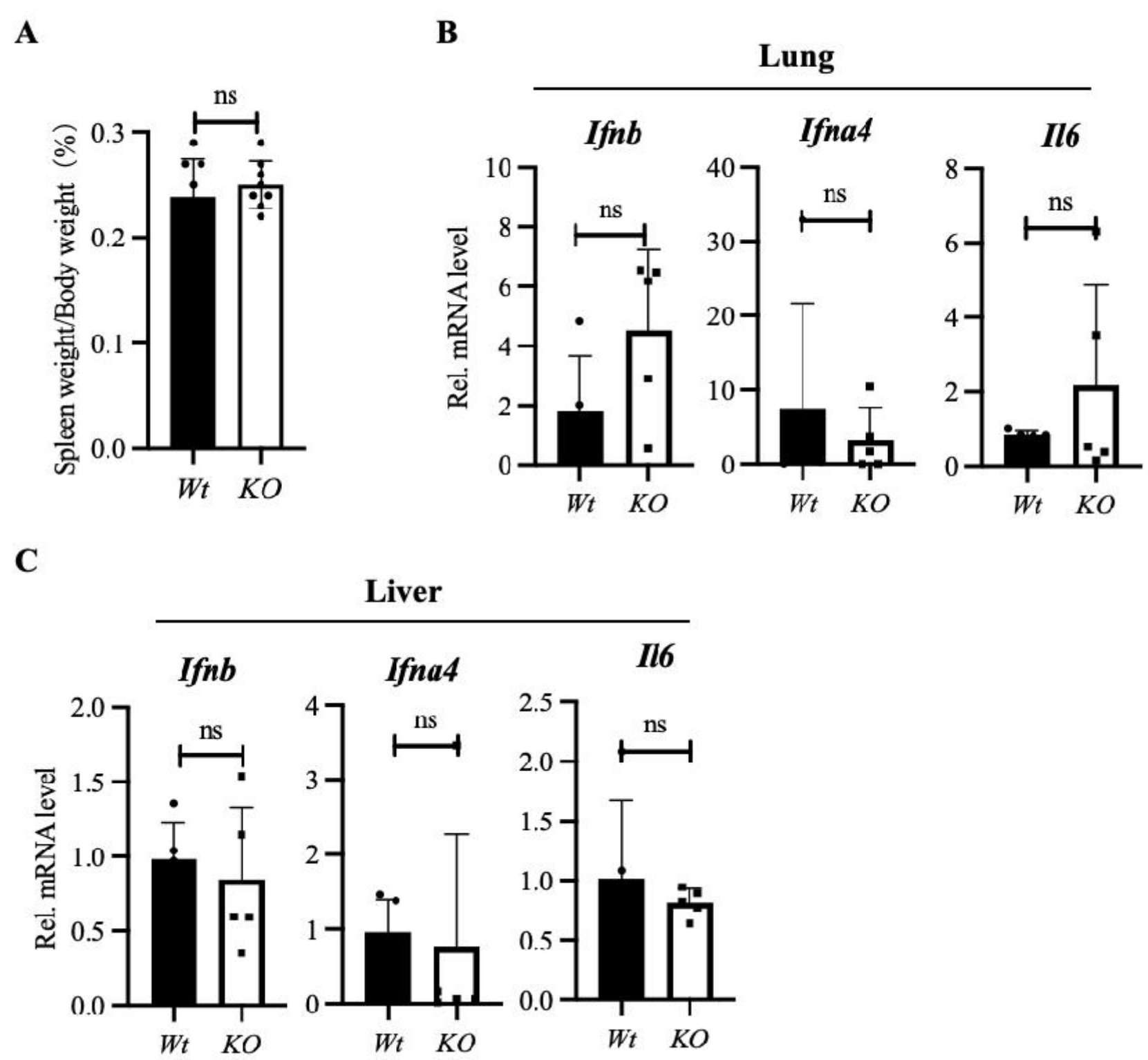

Supplementary Figure 2. DNASE1L3 enhances antiviral innate immune responses *in vivo*. (A) The ratio of spleen weight to body weight of mock-infected (Mock) *Wt* (n = 7) and KO mice (n=8)**. (B-C)** Quantitative RT-PCR analysis on the expression of *Ifnb*, *Ifna4*, *Il6* mRNA in the lungs (B) and livers (C)from mock-infected (Mock)*Wt* and *KO* mice (n = 5 for each group). ns: non-significance. Statistical significance was determined using two-tailed Student's t tests in (A-C).

Figure S3

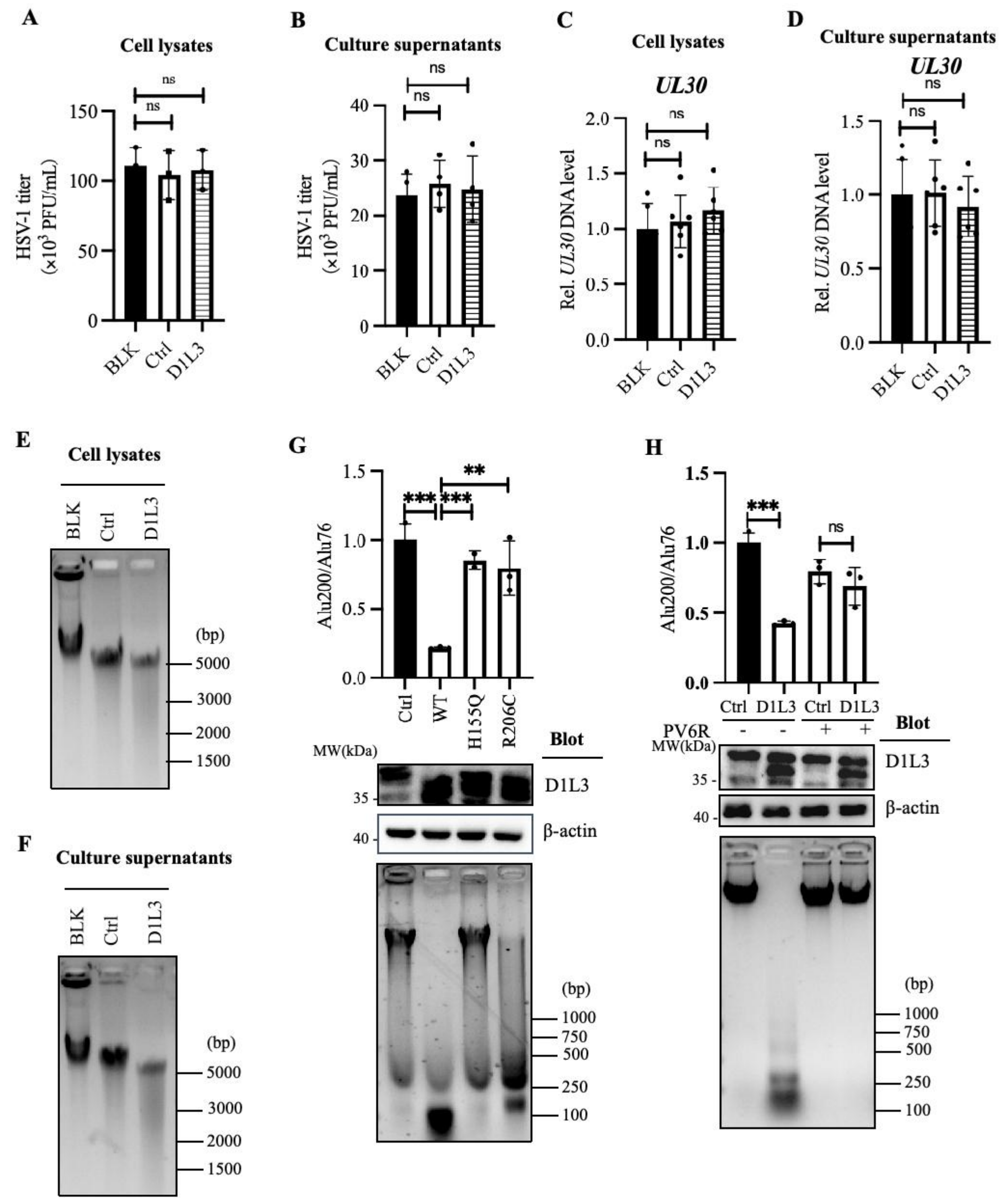

Supplementary Figure 3. Nuclease activity is required for DNASE1L3 to enhance HSV-1-induced innate immune responses.

(A-B) HSV-1 treated with cell lysates(A) or cell culture supernatants(B) from Ctrl or D1L3 THP-1 cells were used for plaque formation assay. Lysis buffer or culture medium treatment serves as blank treatment (BLK). **(C-D)** Purified HSV-1 particles were incubated with cell lysates(C) or cell culture supernatants(D) from Ctrl and D1L3-overexpressing THP-1 cells, and viral abundance was determined by quantitative PCR. Lysis buffer or culture medium treatment serves as blank treatment (BLK). **(E-F)** Isolated nuclei were incubated with cell lysates(E) or cell culture supernatants(F) from Ctrl and D1L3-overexpressing THP-1 cells, followed by agarose gel electrophoresis. Lysis buffer or culture medium treatment serves as blank treatment (BLK). **(G)** Cell lysates from HEK293T cells

transfected with empty vector (Ctrl), wildtype DNASE1L3 (WT), and mutant DNASE1L3 (H155Q and R206C) were used to treat nucleus isolated from 143B cells. Quantitative PCR was performed to measure the ratio of Alu200 to Alu76. Agarose gel electrophoresis analyzed the products digested by cell lysates. Western blotting was performed to confirm the overexpression. **(H)** The cell lysate was collected from HEK293T cells transfected with vector (-), DNASE1L3(D1L3) with Vehicle or PV6R (100 μM) treatment for 6 h. The nucleus for nuclease digestion was obtained from 143B cells. Quantitative PCR was performed to measure the ratio of Alu200 to Alu76. Agarose gel electrophoresis analysis of the products digested by cell lysate. *: $P < 0.05$, **: $P < 0.01$, ***: $P < 0.001$. Statistical significance was determined using one-way ANOVA in (A-D, G-H).

Figure S4

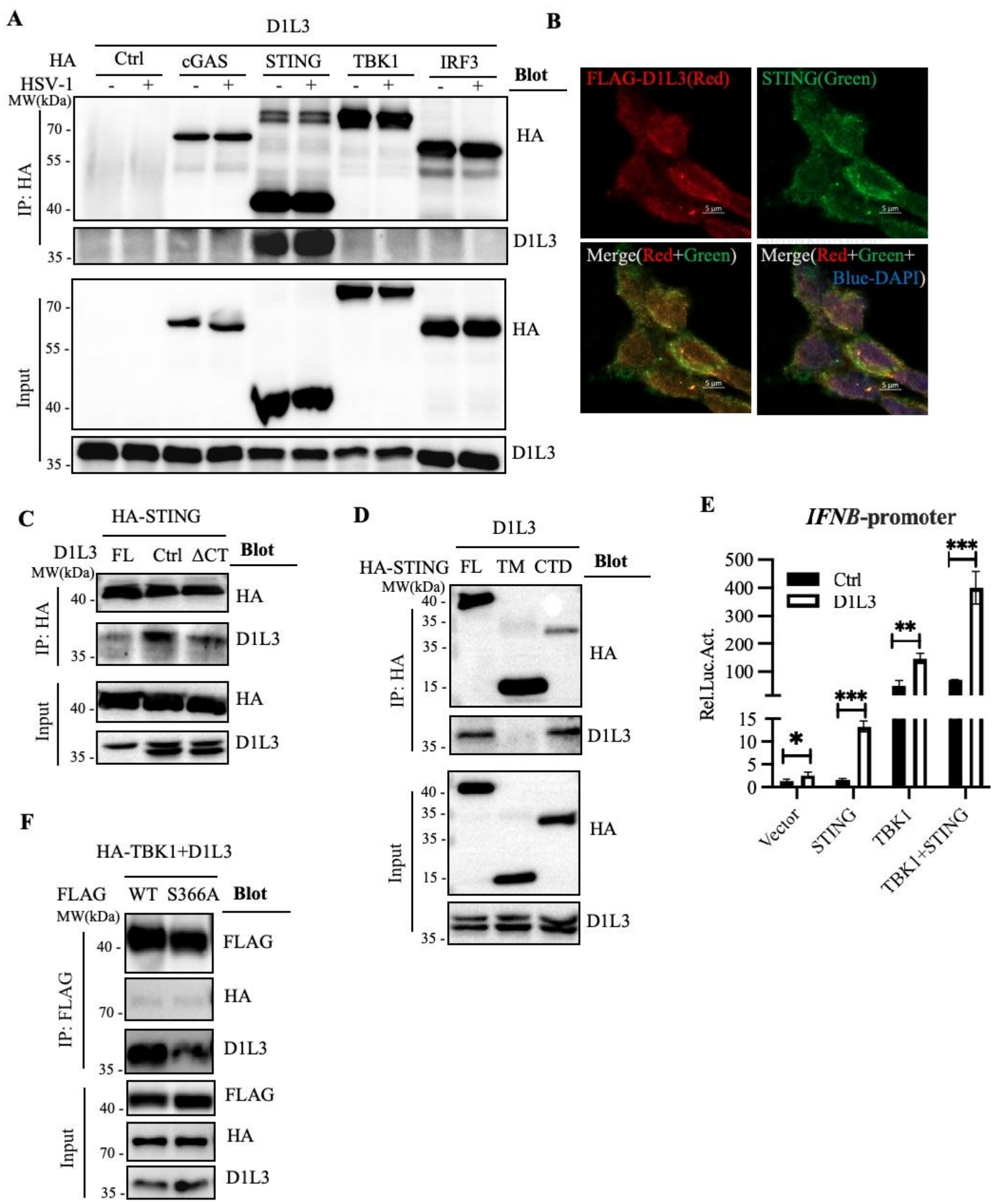

Supplementary Figure 4. DNASE1L3 promotes TBK1-induced STING activation.

(A) Cell lysates from Mock-(-) or HSV-1-infected (+) HEK293T cells transfected with DNASE1L3-expressing plasmid in combination with plasmids expressing different proteins as indicated were used for immunoprecipitation with anti-HA antibody (IP: HA). Western blotting analysis on immunoprecipitation and Input control was performed to measure protein levels as indicated. **(B)** Immunofluorescence imaging the colocalization of FLAG-DNASE1L3 (red fluorescence) and STING (green fluorescence) in HEK293T cells transfected with FLAG-DNASE1L3 and STING. Scale bars represent 5 μm. **(C)** HEK293T cells transfected with HA-STING in combination with full-length DNASE1L3 (FL) or C-terminal truncation DNASE1L3 (ΔCT) followed by Western blotting analysis. **(D)** HEK293T cells transfected with DNASE1L3 in combination with full-length HA-STING (FL), transmembrane domain of HA-STING(TM) or C-terminal domain of HA-STING (CTD) followed by Western blotting analysis. **(E)** The activity of IFNB gene promoter was analyzed by dual luciferase reporter assay in HEK293T cells transfected with different combinations of Vector, STING, TBK1, Ctrl, and D1L3. **(F)** HEK293T cells transfected with HA-TBK1 and D1L3 in combination with WT FLAG-STING or FLAG-S366A STING mutant followed by Western blotting analysis. *: $P < 0.05$, **: $P < 0.01$, ***: $P < 0.001$. Statistical significance was determined using two-way ANOVA.

Figure S5

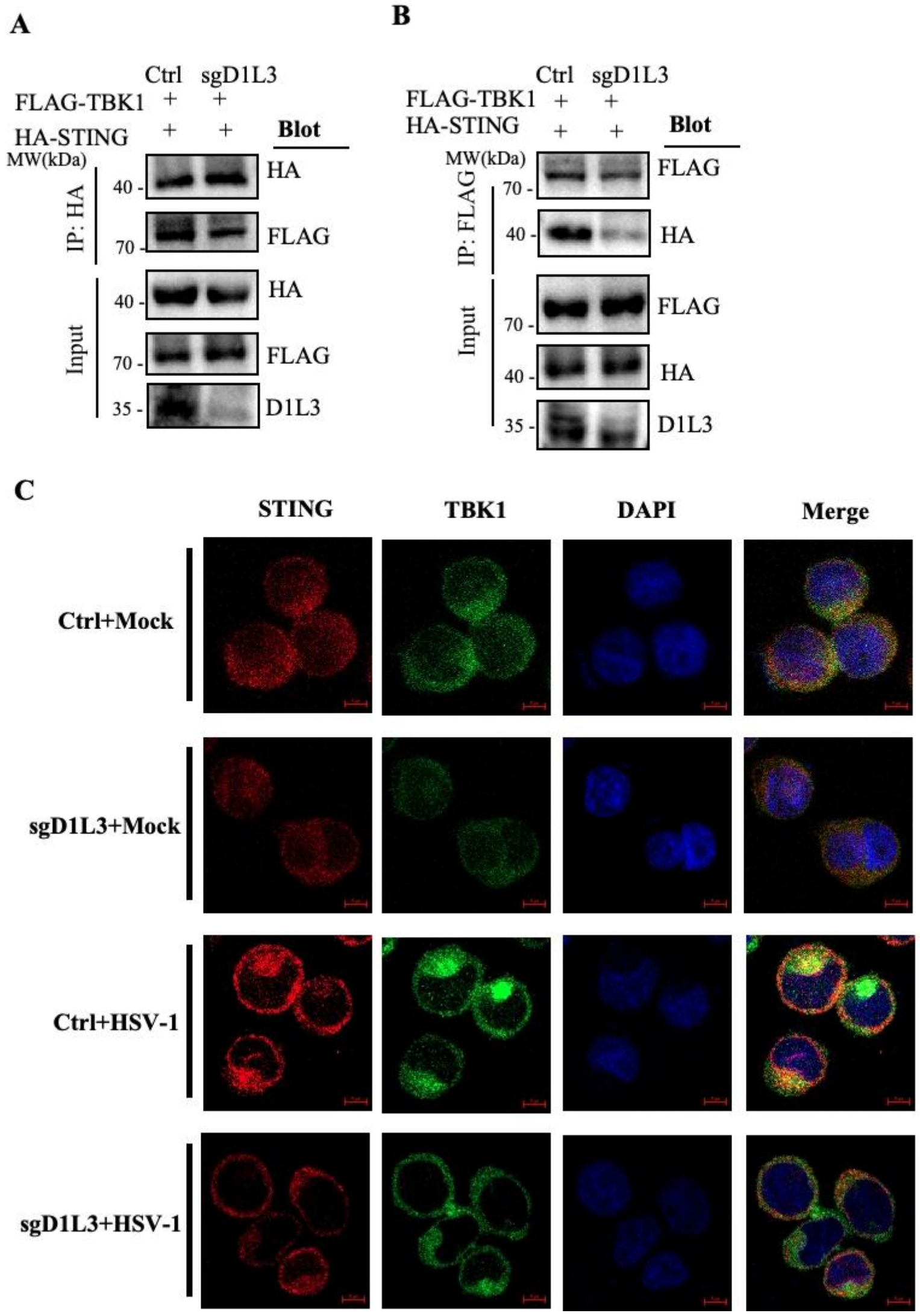

Supplementary Figure 5. DNASE1L3 promotes TBK1-induced STING activation independently of its enzyme activity.

(A) Ctrl and sgD1L3 HEK293T cells were transfected with HA-STING and FLAG-TBK1 expression plasmids. Cell lysates were subjected to immunoprecipitation using an anti-HA antibody, followed by western blot analysis. **(B)**Ctrl and sgD1L3 HEK293T cells were transfected with HA-STING and FLAG-TBK1 expression plasmids. Cell lysates were subjected to immunoprecipitation using an anti-FLAG antibody, followed by western blot analysis. **(C)**Immunofluorescence imaging the co-location of STING (red fluorescence) and TBK1 (green fluorescence) in Mock or HSV-1-infected Ctrl and sgD1L3 THP-1 cells. Scale bars represent 5 μm.

Figure S6

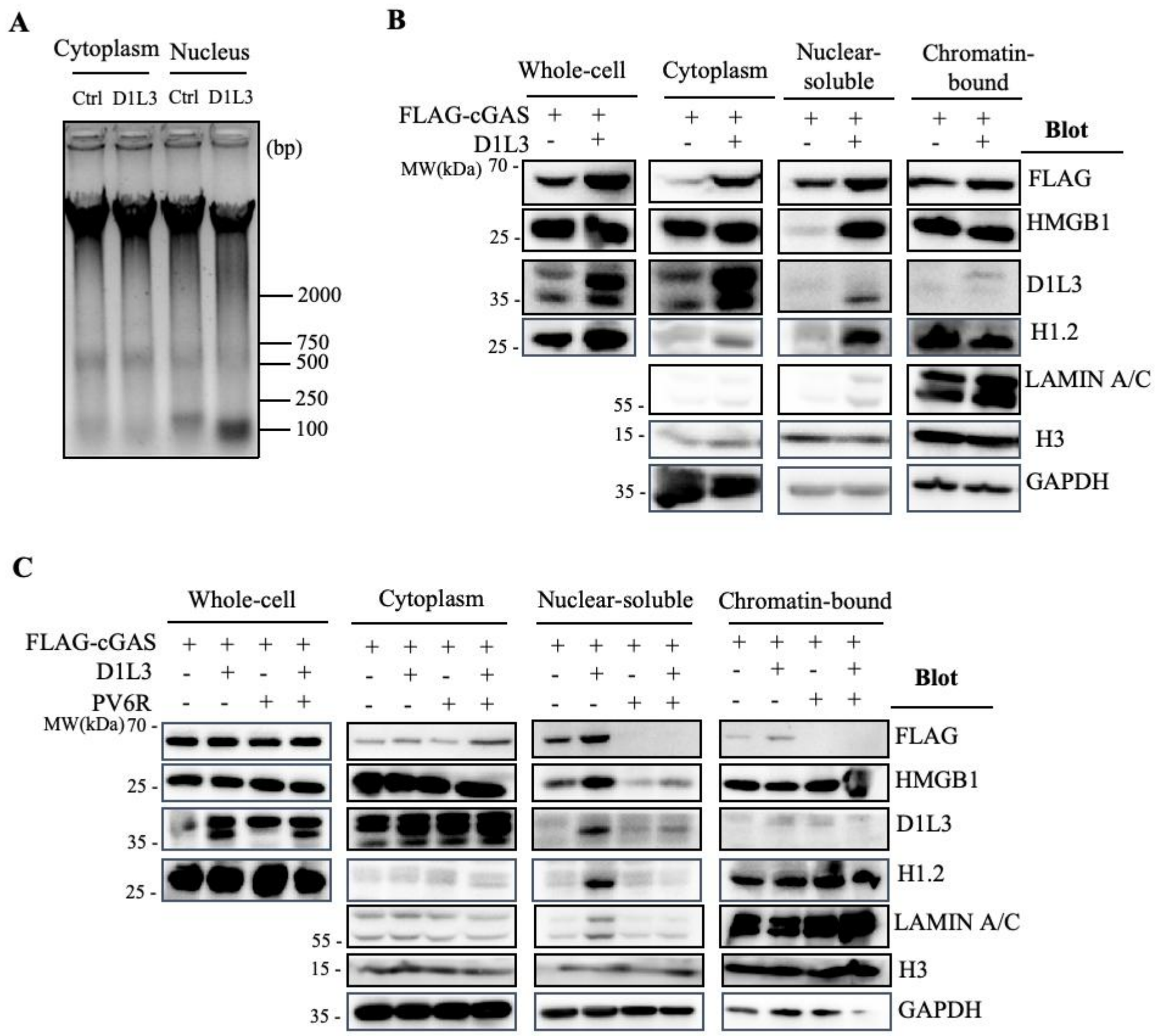

Supplementary Figure 6. DNASE1L3 promotes nucleus release of cGAS, HMGB1, and H1.2. **(A)**This panel shows agarose gel electrophoresis corresponding to the qPCR results in Figure 8A, in which genomic DNA isolated from 143B cells was treated with cytoplasm and nucleus lysates isolated from HSV-1-infected Ctrl and D1L3 THP-1 cells. **(B)** The whole cell lysates as well as cytoplasmic, nucleus-soluble, and chromatin-bound fractions prepared from HEK293T cells that were transfected with FLAG-cGAS in combination with Vector (-) or DNASE1L3(+) were used for Western blotting analysis as indicated. **(C)** HEK293T cells that were transfected with FLAG-cGAS in combination with Vector (-) or DNASE1L3(+) were treated with Vehicle (-) or PV6R (+) and used to prepare the whole cell lysates as well as cytoplasmic, nucleus-soluble, and chromatin-bound fractions followed by Western blotting analysis.

Figure S7

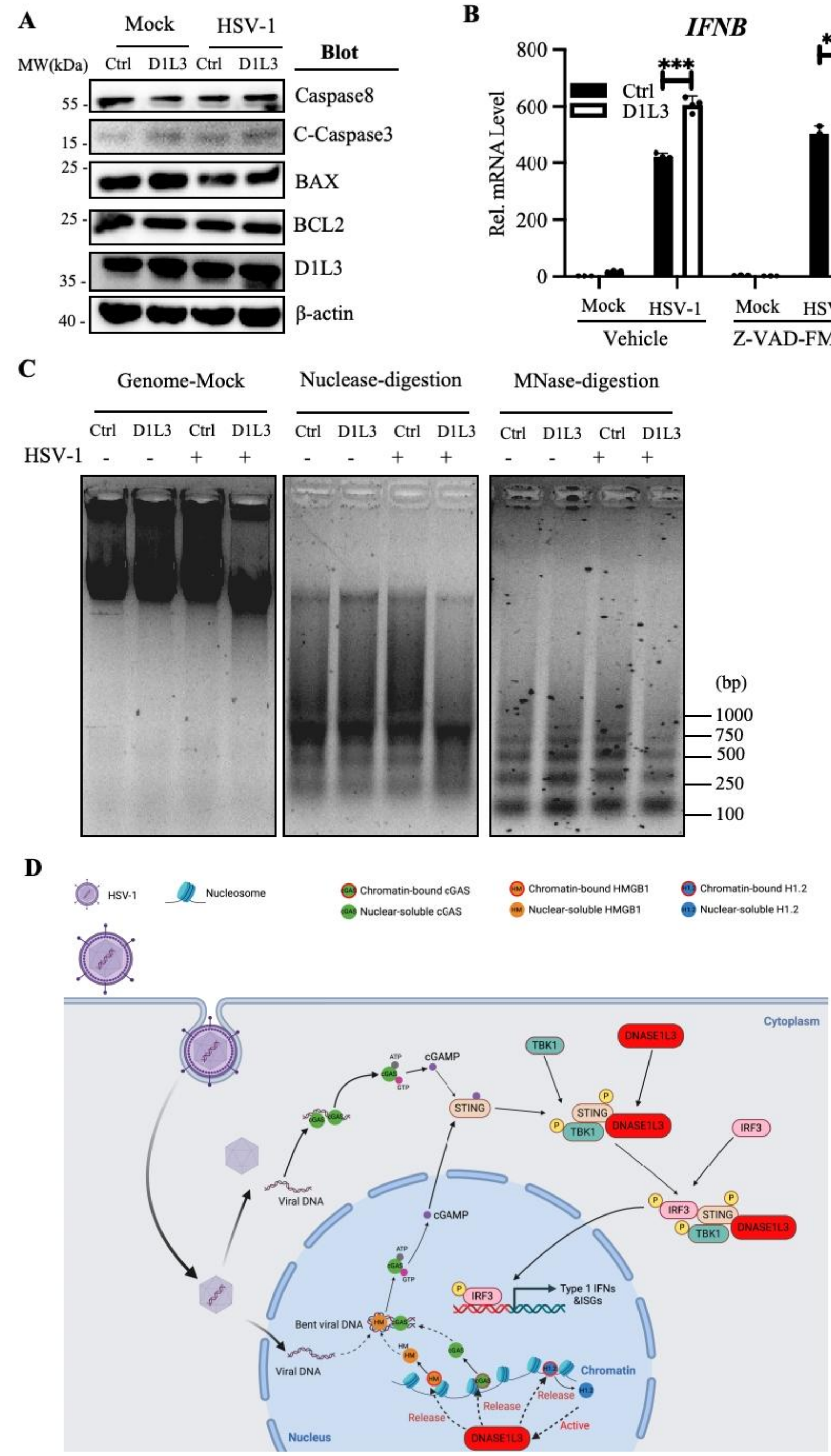

Supplementary Figure 7. DNASE1L3 facilitates HSV-1-regulated chromatin accessibility.

(A) Western blotting analysis of apoptosis-related indicators Caspase8, Cleaved-Caspase3 (C-Caspase3), BAX, BCL2 in Ctrl or D1L3 THP-1 cells infected with HSV-1. **(B)** Quantitative PCR analysis of *IFNB* mRNA in Ctrl or D1L3 THP-1 cells infected with HSV-1, and treated with Vehicle or Z-VAD-FMK (5 μM). *: $P < 0.05$, **: $P < 0.01$, ***: $P < 0.001$(two-tailed Student's t-test). **(C)** Ctrl and D1L3 cells of THP-1 were infected with HSV-1 for 4h, and cell samples were collected and divided equally into triplicate. One sample was fixed with formaldehyde, then the nuclei were

extracted and digested with protease K to obtain genomic DNA samples (Genome-Mock). One sample was used to isolate the nucleus, and the reaction was performed at 37℃ for 10 min to allow DNASE1L3 in the nucleus to cleave its own genome, and then digested with protease K to obtain DNA samples (Nuclease-digestion). One sample was fixed with formaldehyde and used to isolate the nucleus, MNase was added to the reaction at 37℃ for 10 min, and then digested with protease K to obtain DNA samples (MNase-digestion). All DNA samples were subjected to gel electrophoresis. **(D)** A model of DNASE1L3-mediated cGAS-STING activation via dual mechanisms during HSV-1 infection.

2.2 Supplementary Tables

Supplementary Table 1. Sequences of primer sets for qRT-PCR.

Gene	Species	Forward	Reverse
β-actin	Mus	GGCTGTATTCCCCTCCATCG	CCAGTTGGTAACAATGCCATGT
Dnase1l3		CTCTGCTCCTTCAATGTGAGG	GCTGCTGTCCTTGATTTCCAT
Ifna4		GCCTTGACAGTCCTGGAAGA	GGATCTGTTGGTTATCCACC
Dnase1		ATGCGGTACACAGGGCTAATG	AAAAGTCCGAATGTTGAAGGCT
Dnase1l1		ATGCCCTTTGGACAACCTGG	GAGCAGGGGAGCCATAACAAG
Dnase2a		AAGCCCTGAGCTGCTATGG	ATACGTCAGTCCCTTTGGAGTA
Dnase2b		CCCAAAAGGACTAGCAAGGCA	TCCAGGTTTGTCTTGTGGAGT
Pld3		AAGCCCAAACTGATGTACCAG	CCTTCCATGCCTCGATTTCATT
Dnase1l2		GCGGATTGGAGCCTTCAATGT	GTCTGGGTCTCGTACCTCTTG
Trex1		CAGACCCTCATCTTCTTAGACCT	CAGGGCTACAGGCTTTCCC
Samhd1		TGCAGACGACGACTTCCAAAA	TCTCGGAAACCACGATTCTCTAA
Il6		CTGCAAGAGACTTCCATCCAG	AGTGGTATAGACAGGTCTGTTGG
Tnfa		CAGGCGGTGCCTATGTCTC	CGATCACCCCGAAGTTCAGTAG
Ifnb		TTGACGTGGGAGATGTCCT	ATCCAGGCGTAGCTGTTGTA

Alu-76	Homo	AGACCATCCTGGCTAACACG	GTTCACGCCATTCTCCTGC
Alu-200		AAAATTAGCCGGGCGTG	AGACGGAGTCTCGCTCTGTC
β-actin		CTCCATCCTGGCCTCGCTGT	GCTGTCACCTTCACCGTTCC
IFNB		GCTTGGATTCCTACAAAGAAGCA	CAAAGTTCATCCTGTCCTTGAGG
TNFA		CCTCTCTCTAATCAGCCCTCTG	GAGGACCTGGGAGTAGATGAG
IL6		TCATCACTGGTCTTTTGGAG	GTCAGGGGTGGTTATTGC
DNASE1L3		GCGACCACTTTCCAGTTGAA	CTTGGGTCTAGGAGCGTTTG
DNASE2A		GCCAGCTCTTAGAGGGTCC	CTCCGGGCTGTTGATGAGT
DNASE1		GCCGTGTCCCTGAAGATCG	TGGTGCATCCTGATTGAGGTT
DNASE1L1		CTGCACACCACTCCTAAGGC	CAGGCGCTTTTTGGTCAGT
DNASE2B		GCTCAACGGCTGAAGACACA	GGACTCCGATTTAGGTCTCCAAT
PLD3		AAGCCTAAACTGATGTACCAGGA	GCCTCAATCTCATTCATGGGC
DNASE1L2		TGTCCGAGCACGAGTACAG	GCCGAGAACTTGACCACGAA
TREX1		CGCATGGGCGTCAATGTTTT	GCAGTGATGCTATCCACACAGAA
SAMHD1		CTGGAACTCCATCCCGACTAC	AGTAATGCGCCTGTGATTTCAT
ICP0	HSV-1	CCTGTCGCCTTACGTGAACA	CCATGTTTCCCGTCTGGTCC
UL30		TTACTTCTCCCACCTGTTGG	GCCACACTTCGGGAATAAAC

Supplementary Table 2. Sequences of primer sets for sgRNA constructs

Gene	Number	Forward	Reverse
DNASE1L3	sgRNA1	caccGAGCAGCGCCAGGATGTCAC	aaacGTGACATCCTGGCGCTGCTC
	sgRNA2	caccGTCCTCATGGCCAGGGCGCTG	aaacCAGCGCCCTGGCCATGAGGAC
	sgRNA3	caccGCTTCAACGTCAGGTCCTTTG	aaacCAAAGGACCTGACGTTGAAGC
	sgRNA4	caccGCAGGAAGACAAGAATGCCA	aaacTGGCATTCTTGTCTTCCTGC
